# Substance use disorder and altered hemispheric asymmetries: A systematic review

**DOI:** 10.1016/j.cpr.2025.102658

**Published:** 2025-10-03

**Authors:** Annakarina Mundorf, Hicret Atilgan, Lisa Deneke, Sebastian Ocklenburg

**Affiliations:** a ISM Institute for Systems Medicine and Department of Human Medicine, MSH Medical School Hamburg, Hamburg, Germany; b Department of Neurology, Division of Cognitive Neuroscience, Johns Hopkins University School of Medicine, Baltimore, MD, USA; c Department of Psychology, Medical School Hamburg, Hamburg, Germany; d ICAN Institute for Cognitive and Affective Neuroscience, Medical School Hamburg, Hamburg, Germany; e Institute of Cognitive Neuroscience, Biopsychology, Faculty of Psychology, Ruhr University Bochum, Bochum, Germany

**Keywords:** Lateralization, Handedness, Alcoholism, Dependence, Substance abuse, Neuroimaging, Hemispheric asymmetries

## Abstract

Substance use disorder (SUD) is characterized by compulsive use despite adverse consequences and may be influenced by brain asymmetry affecting cognitive and emotional processes. This systematic review investigates the relationship between brain asymmetry and SUD. PubMed, Web of Science, and PsycInfo were searched for articles published until July 2025, using the search terms: ((Alcoholism) OR (alcohol abuse) OR (substance abuse) OR (addiction)) AND ((handedness) OR (footedness) OR (dichotic listening) OR (line bisection task) OR (visual half field technique) OR (fMRI asymmetry) OR (EEG asymmetry) OR (structural asymmetry)). Inclusion criteria were (i) subjects having a diagnosis of or meeting the criteria for alcoholism, alcohol abuse, substance abuse, or addiction assessed with a validated clinical inventory, (ii) articles must contain information on handedness, footedness, dichotic listening, line bisection task, the visual half-field technique, or hemispheric differences (iii) data must be given for the clinical group separately, (iv) original research article in the English language. For neuroimaging studies, both hemispheres needed to be examined separately. Exclusion criteria included: (i) review articles; (ii) studies without matched groups; (iii) studies on recreational use only; (iv) those involving prenatal substance exposure or comorbid neurological disorders. Risk of bias was assessed with the Newcastle-Ottawa Scale. Forty-nine studies met the criteria. Structural imaging indicates asymmetric white and grey matter alterations: reduced left-hemispheric white matter integrity and lower grey matter volume in frontal and temporal regions. Functional data show compensatory right-hemispheric activation. Behavioral lateralization findings vary by substance type, sex, and age, with potential implications for personalized treatment strategies.

## 1. Introduction

Substance use disorder (SUD) is a complex phenomenon characterized by a compulsive pattern of use despite adverse consequences, and it poses significant challenges for individuals and society alike (American Psychiatric Association, 2013). It is estimated that around 2–5 % of the global population engages in the use of drugs, with a significant portion experiencing some form of SUD (Shen et al., 2023). SUD is characterized by an individual’s inability to control their use of substances despite facing negative consequences. It includes a range of behaviors and symptoms associated with the misuse of alcohol, drugs, or other substances. According to the Diagnostic and Statistical Manual of Mental Disorders (DSM-5), SUD is diagnosed based on specific criteria. These include taking the substance in larger amounts or for a longer period than intended, unsuccessful attempts to cut down or control its use, and spending significant time obtaining, using, or recovering from the substance. Individuals may experience cravings, fail to meet obligations at work or home, and continue using substances despite social or interpersonal issues. Other indicators include giving up important activities, using substances in hazardous situations, developing tolerance, and experiencing withdrawal symptoms. Among the most consumed substances are alcohol, nicotine, heroin, and methamphetamine (American Psychiatric Association, 2013).

Understanding the complexity of SUD involves examining not only the behavioral and psychological aspects but also the underlying neurobiological mechanisms. Studies suggest that differences in structural and functional hemispheric asymmetries may play a crucial role in modulating various cognitive and emotional processes associated with SUD (Cao et al., 2021; Mundorf et al., 2021; Mundorf & Ocklenburg, 2021). On the structural level, researchers examined 38 SUD subjects and 19 healthy controls, focusing on alcohol. They found decreased fractional anisotropy (FA) in the left external capsule and superior longitudinal fasciculus in the SUD group, indicating altered white matter integrity associated with alcohol dependence (Chumin et al., 2018). Others analyzed 19 individuals with SUD and 20 healthy controls for differences in global grey matter asymmetry, revealing no overall differences but specific regional brain changes related to alcohol use (Zhu et al., 2018). In another study focusing on 20 cocaine-dependent individuals and 16 healthy controls, a significant reduction in grey matter volume in the left striatum and right supramarginal gyrus was evident among SUD participants, which suggests that cocaine dependence is linked to specific structural brain alterations (Barrós-Loscertales et al., 2011). In 2021, the ENIGMA Addiction Working Group analyzed cortical and subcortical asymmetries in substance dependence and found that substance dependence is significantly associated with differences in volume asymmetry of the nucleus accumbens, showing a less pronounced rightward asymmetry. This effect was particularly evident in individuals with alcohol or nicotine dependence, suggesting that these conditions are linked to reduced rightward volume asymmetry compared to control subjects (Cao et al., 2021).

On the functional level, reviewing the literature on impulsivity, substance craving, and left and right hemisphere activation revealed that most activation peaks favored the right hemisphere (Gordon, 2016). However, there was a left hemisphere dominance by 6.7 % for cue-induced craving. This left asymmetry was consistently observed for alcohol, cocaine, and heroin. In the case of nicotine, only deprived individuals exhibited left-hemisphere activation similar to other substances, while satiated smokers displayed a rightward asymmetry (Gordon, 2016). Others investigated 27 tobacco-dependent individuals and 25 healthy controls using resting-state functional magnetic resonance imaging (fMRI). Results showed decreased connectivity in the right caudate and bilateral anterior cingulate cortex (ACC), with increased activity in the right caudate correlating with cravings, indicating altered brain regions associated with tobacco use (Rogers et al., 2012). In a larger sample of 193 SUD patients and 108 controls focusing on alcohol, researchers utilized electroencephalography (EEG) to reveal decreased left relative to right frontal alpha band power in the SUD group (Hayden et al., 2006). This suggests a significant shift in brain activity patterns linked to alcohol dependence, contrasting with the structural findings of Rogers et al. (2012).

Over 90 % of cortical and subcortical regions exhibit structural asymmetry (Guadalupe et al., 2017; Kong et al., 2022). For instance, many individuals display leftward volume asymmetry in the planum temporale, with language processing primarily lateralized to the left hemisphere. The right hemisphere is associated with spatial orientation, attention, and extralinguistic communication (Vingerhoets, 2019). These inherent asymmetries are a fundamental organizational principle across species, contributing to efficient brain function (Ocklenburg & Güntürkün, 2024) and demonstrating alteration in several psychiatric and neurological conditions (Mundorf et al., 2021; Mundorf & Ocklenburg, 2021; Ocklenburg et al., 2024).

Hemispheric asymmetries are also reflected in behavioral asymmetries, with the most frequently assessed form being handedness, i.e., the preference of one hand over the other for specific tasks (Ocklenburg & Güntürkün, 2024). In subjects with SUD, altered prevalences in behavioral lateralization have been found, depending on the substance used. Early studies report increased prevalences of left and non-right-handedness of 15 % to 39 % and 25 %, respectively, when assessing writing hand preferences (Bakan, 1973; Smith & Chyatte, 1983). Sperling (2000) reported increased rates of non-right-handedness up to 44 % in males affected by alcohol-induced SUD (Sperling, 2000). However, others with equal sample size failed to find significant differences in handedness prevalence between SUD subjects and controls (Weinland et al., 2019). Notably, in the general population, approximately 80 % to 90 % of individuals are right-handed, while the prevalence of left-handedness ranges from 9.3 % - based on strict criteria for assessing left-handedness - to 18.1 % when considering a broader definition of non-right-handedness, which includes those who prefer the left hand or use both hands with equal frequency (Papadatou-Pastou et al., 2020).

Other categories include footedness, eyedness, and earedness (Mundorf et al., 2023; Ocklenburg & Güntürkün, 2024). For instance, subjects with heroin addiction showed increased right foot preference similar to controls, whereas subjects with alcohol-induced SUD had no side preference (Mandal, 2000). The same research also revealed that participants with heroin-induced SUD displayed a greater inclination toward the right ear and eye, mirroring the control group’s trend, yet subjects with alcohol-induced SUD maintained an absence of any overall side preference (Mandal, 2000).

Functional lateralization can also be assessed via visuospatial attention or language lateralization with the dichotic listening paradigm. For example, Herzig et al. (2010) investigated visuospatial attention with a lateralized lexical decision and a lateralized facial decision task and observed increased right-sided bias in nicotine-dependent subjects compared to controls in both tasks (Herzig et al., 2010).

Based on common research topics in research on lateralization in SUDs, we subdivided the neuroimaging part of the result sections into five distinct thematic parts: Reward Processing, Cognitive Control and Memory, Emotion and Salience, Visual and Sensorimotor Processing, and Global Connectivity. This subdivision aims to enhance readability and allow readers to easily locate information relevant to specific domains. Structural findings were assigned to each section according to the primary functional domain implicated by their results. Similarly, the behavioral findings are organized into four distinct thematic sections: Eyedness and behavioral markers of visuospatial attention and visual perceptual asymmetries, Hemispheric asymmetries for acoustic stimuli, Handedness, and Footedness.

Taken together, the literature on hemispheric asymmetries and SUD is methodologically heterogeneous and shows a variety of result patterns regarding the prevalence of atypical asymmetries in SUD. A systematic review exploring and synthesizing these findings is currently missing from the literature. This, however, is crucial since the interplay between hemispheric asymmetry and addiction vulnerability invites a deeper exploration of how neurological factors contribute to the development and maintenance of substance dependence, highlighting potential avenues for targeted interventions and therapeutic approaches. This work thus aims to systematically review the evidence on the relationship between brain asymmetry and SUDs, focusing on how structural and functional differences, along with behavioral factors, influence cognitive and emotional processes in individuals with substance dependence. By analyzing relevant neuroimaging and behavioral studies, we seek to enhance our understanding of the neurological and psychological foundations of addiction and inform future interventions.

## 2. Methods

A systematic literature review was conducted following Prisma Guidelines (Page et al., 2021) in the databases Web of Science (https://www.webofscience.com/wos/woscc/basic-search), PubMed (https://pubmed.ncbi.nlm.nih.gov/) and PsycInfo (https://search.ebscohost.com/) for studies published until July 2025. The databases were searched with the term: ((Alcoholism) OR (alcohol abuse) OR (substance abuse) OR (addiction)) AND ((handedness) OR (footedness) OR (dichotic listening) OR (line bisection task) OR (visual half field technique) OR (fMRI asymmetry) OR (EEG asymmetry) OR (structural asymmetry)). The search term was selected to identify studies explicitly examining hemispheric asymmetry in SUD. Both studies reporting formal asymmetry indices and those presenting unilateral hemisphere-specific findings without direct asymmetry comparison were included. No automation tools were used. Identifying relevant studies involved several steps, starting with the screening based on the titles and abstracts of the retrieved articles. Inclusion criteria were (i) subjects either having a diagnosis of or meeting the criteria for alcoholism, alcohol abuse, substance abuse, or addiction assessed with a validated clinical inventory, (ii) articles must contain information on either handedness, footedness, dichotic listening, line bisection task, the visual half-field technique, or hemispheric differences (iii) data must be given for the clinical group separately, (iv) original research article in the English language. For neuroimaging studies, only those that examined both hemispheres separately to assess asymmetry were included. We included both studies that conducted direct asymmetry comparisons (e.g., asymmetry indices or statistical tests between hemispheres) and those that reported hemisphere-specific results (e.g., left and right activation or structural values), even if no formal asymmetry analysis was performed. Studies without direct asymmetry comparisons were considered relevant when the reported data allowed for qualitative or indirect inferences about hemispheric lateralization (e.g., separate reporting of left and right hemisphere values without statistical comparison). For missing data on handedness, corresponding authors were contacted. Exclusion criteria were (i) review articles, (ii) no matching of clinical and control groups based on side preference for behavioral studies, (iii) subjects of experimental group not meeting the criteria for alcoholism, alcohol abuse, substance abuse, or addiction, (iv) solely familiar consumption, (v) prenatal substance exposure, or (vi) comorbid neurological disorders. Two independent raters conducted all steps to ensure a reliable and unbiased selection of studies. Conflicts were resolved by consensus or through a third-party reviewer at all stages. The study was not pre-registered, no review protocol was prepared and Risk of Bias was assessed with the Newcastle Ottawa Scale (NOS) for non-randomized, non-comparative intervention studies (Wells et al., 2011), see Table S3.

## 3. Results

### 3.1. Study selection

**Fig. 1.**
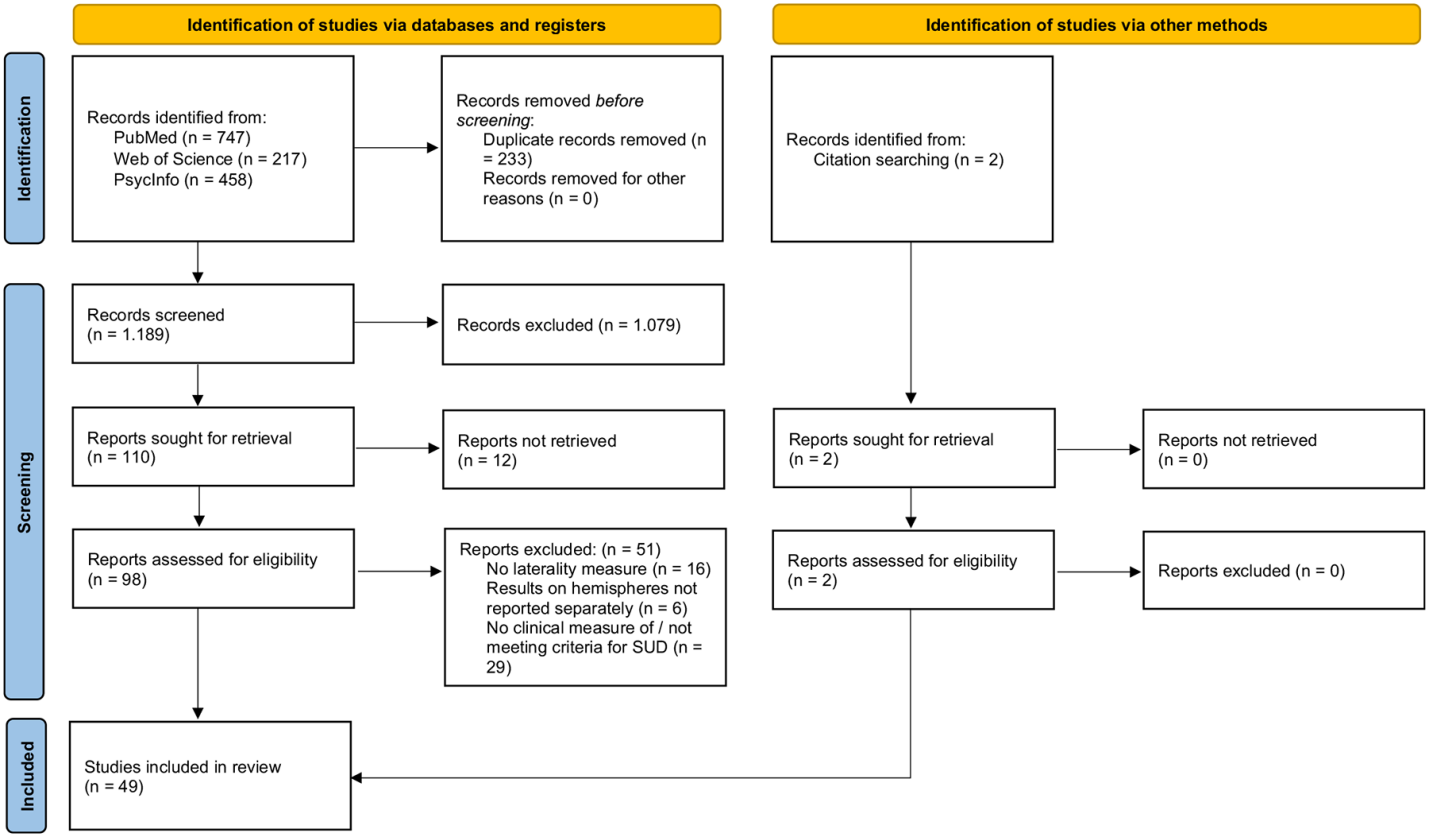
PRISMA Flow diagram depicting the process of identifying, screening, and inclusion of the literature. Adapted from Page et al. (2021).

One hundred reports were assessed for eligibility following the inclusion and exclusion criteria (98 via identification of studies via databases and registers, and two via citation searching), leading to the inclusion of 49 studies and the exclusion of 51 studies after the full-text screening. Reasons for exclusion were: No laterality measure (*n* = 16), results on hemispheres not reported separately (*n* = 6), and no clinical measure of / not meeting criteria for dependence or SUD (*n* = 29). The full process is presented in the flow chart diagram following Prisma Guidelines in Fig. 1, adapted from (Page et al., 2021) and in the PRISMA checklists (Tables S1 and S2).

Three studies appeared suitable for examining hemispheric coordination, but they do not specifically measure hemispheric asymmetry (Al-Khalil et al., 2021; Cao et al., 2023; Clark et al., 2007). Similarly, Dai et al. (2021) employ a mirror-symmetric approach, which assumes functional symmetry between homologous regions in each hemisphere. As our focus is on exploring hemispheric differences, we exclude these studies from the review in favor of those that explicitly investigate asymmetries between the hemispheres. While Meiers et al. (2020) screened adolescents using the Alcohol Use Disorder Identification Test, the results primarily reflect a sample with minimal indications of alcohol use disorder, as only one participant (2 %) exceeded the clinical cutoff. As the focus of this review is on SUD, we excluded this study in favor of research that specifically examines more pronounced addictive behaviors. Similarly, exclusion criterion 3 was met for 14 % of the experimental group in the study by Schermitzler et al. (2025) and for 40 % in the study by Smith et al. (2014). This careful selection process ensured that only studies providing relevant and direct insights into hemispheric lateralization were included in the review.

### 3.2. Results of risk of bias analysis

All but nine studies scored six or more points on the scale, indicating a low risk of bias (notably, the maximum possible score was eight, as the “validity of non-response rate” was not applicable; see Table S3). Studies with a high risk of bias (< 5 stars) are included for completeness and are highlighted in the text.

### 3.3. Neuroimaging studies

This review includes 34 studies reporting findings on hemispheric asymmetries and functional lateralization via neuroimaging and one behavioral study reporting findings from visuospatial attention in SUD. Structural asymmetries were measured with MRI, diffusion tensor magnetic resonance imaging (DTI), or voxel-based morphometry (VBM). Functional lateralization was assessed with fMRI or EEG. The results are organized into five thematic subheadings: Reward Processing, Cognitive Control and Memory, Emotion and Salience, Visual and Sensory-Motor, and Global Connectivity. Each section presents relevant findings on structural and/or functional asymmetries, including results from both resting-state and task-based paradigms where applicable. To facilitate differentiation between studies that formally assess hemispheric asymmetry and those reporting unilateral or hemisphere-specific findings without a formal comparison, the tables include a column categorizing studies based on their approach to assessing asymmetry. Unless specified otherwise in the text, the subjects were adults.

#### 3.3.1. Reward processing

Reward processing is consistently disrupted in individuals with SUD, particularly within frontostriatal and mesolimbic pathways (Volkow & Boyle, 2018). These systems, including the ventral striatum, nucleus accumbens, and orbitofrontal cortex, exhibit well-documented hemispheric asymmetries in healthy individuals (Budilin et al., 2008; Cao et al., 2021; Kong et al., 2022; Korponay et al., 2022). Such asymmetries may be relevant for understanding the neural basis of addiction-related behaviors, including craving and compulsive drug-seeking. The following studies examine how structural and functional asymmetries in reward-related brain regions are altered in individuals with SUD. See Table 1 for an overview of the studies included in this section.

Balconi et al. (2014) observed asymmetries in reward processing in an Iowa Gambling Task in forty cocaine SUD subjects (23 men) and forty-two (24 men) controls. The behavioral responses (gain/ loss options) and lateralized alpha band modulation (more precisely: frontal brain log-transformed asymmetry for mean power) were assessed for the analysis. Compared to controls, SUD subjects demonstrated increased left-hemispheric activation in response to losing with direct reward, suggesting possible dysregulation in left-lateralized reward circuits associated with maladaptive decision-making (Balconi et al., 2014).

Costumero et al. (2017) investigated how functional brain networks in cocaine-dependent individuals are modulated by non-drug rewarding stimuli, specifically erotic images. Using independent component analysis (ICA) on fMRI data, researchers examined the modulation of functional networks in 20 abstinent cocaine-dependent male individuals compared to 21 healthy male controls. The results revealed that cocaine-dependent participants exhibited reduced modulation of the left frontoparietal network (FPN) in response to unexpected erotic stimuli. Furthermore, longer periods of abstinence were associated with greater modulation of this network, suggesting a potential recovery of reward processing over time. The authors concluded that these findings align with addiction models proposing that drug dependence reduces sensitivity to non-drug rewards, particularly in cognitive and attentional processes (Costumero et al., 2017). Of note, in a follow-up study, Costumero et al. (2018) further explored the role of the left FPN in cocaine dependence, building upon their earlier findings on reduced FPN modulation in response to non-drug rewards. Here, they examined brain activity in fifteen abstinent cocaine-dependent men and fifteen healthy male controls, using fMRI while the participants viewed cocaine-related, erotic, aversive, and neutral images. While their 2017 study demonstrated that cocaine-dependent individuals exhibited diminished FPN engagement when processing unexpected erotic stimuli, their 2018 study revealed the opposite pattern when participants were exposed to cocaine-related cues. Specifically, cocaine-dependent individuals showed increased activation in the left FPN in response to drug-related images, with this heightened activation positively correlated with the duration of cocaine use. This heightened activation positively correlated with the duration of cocaine use, indicating a lateralized shift in attentional and salience processing. Together, these studies suggest an imbalance in left-lateralized FPN responsiveness: reduced for non-drug rewards, amplified for drug-related cues (Costumero et al., 2017, 2018).

Barrós-Loscertales et al. (2011) assessed structural differences between 20 cocaine-dependent men and 16 healthy control males. Whole brain voxel-wise analyses indicated a decrease in grey matter volume in the left striatum (14.8 %) and the right supramarginal gyrus (12.1 %) in cocaine-dependent men compared to controls (Barrós-Loscertales et al., 2011). These lateralized reductions may reflect structural vulnerability in reward and attention networks.

Tapert et al. (2003) divided 30 adolescents aged 14–17 into two groups: 15 with AUD and 15 non-abusing controls. Using fMRI, participants viewed personalized images of alcoholic and non-alcoholic beverages while completing a task that involved identifying the presence of people in the images. The results showed that the AUD group exhibited significantly greater activation throughout the brain, particularly in the left hemisphere, including the frontal and limbic regions in response to alcohol images compared to the control group. Notably, the ventral ACC, prefrontal cortex (PFC), orbital gyrus, subcallosal cortex, inferior frontal gyrus, paracentral lobule, parahippocampus, amygdala, fusiform gyrus, temporal lobe, hypothalamus, posterior cingulate, precuneus, cuneus, and angular gyrus demonstrated increased activation. Conversely, the control group displayed greater activation in the right middle and inferior frontal regions. This heightened response was linked to increased reported cravings and alcohol consumption. The study suggests that alcohol cues trigger more extensive activation in these left hemisphere regions among adolescents with AUD, indicating a lateralized neural response pattern associated with substance use (Tapert et al., 2003).

Beylergil et al. (2017) examined the neural mechanisms underlying impaired behavioral adaptation in alcohol-dependent patients using fMRI and a reward-guided decision-making task. The study involved 34 abstinent alcohol-dependent patients and 26 age-matched healthy controls, all of whom were male. The findings revealed that patients exhibited reduced sensitivity to punishments and a weaker association between prediction errors (PEs) and dorsolateral PFC (dlPFC) activity, particularly with negative PEs. Notably, there was decreased activity in the left dlPFC of patients during negative PEs, indicating compromised cognitive flexibility and adaptation. The right dlPFC activity in alcohol-dependent subjects showed a reduced correlation with positive PEs, suggesting an impairment in initiating actions to select options that transitioned from being punishing to rewarding after a contingency reversal. Additionally, a positive correlation between punishment sensitivity and right anterior insula activity was observed, indicating impaired detection of punishment events in patients. A positive correlation between punishment sensitivity and right anterior insula activation further supports a lateralized disruption in salience and learning processes (Beylergil et al., 2017).

Sullivan et al. (2005) measured bilateral volumes of the caudate nucleus, putamen, nucleus accumbens, and medial septal/diagonal band in 25 men with alcohol dependence (19 abstinent) and 51 age-matched control men. The analyses revealed no hemispheric difference in volume between groups in all regions (Sullivan et al., 2005). This null finding underscores variability in lateralization effects across reward-relevant structures and substances.

Feng et al. (2016) compared 25 healthy male nonsmokers with 27 age-matched daily male smokers (mean age 20.7) using resting-state fMRI to investigate neural abnormalities and cognitive control deficits in young adult smokers. Participants completed a color-word Stroop task to evaluate cognitive control. The results indicated that smokers exhibited increased fractional amplitude of low-frequency fluctuation in the right caudate, which was positively correlated with craving scores (Feng et al., 2016). Additionally, smokers showed reduced resting state functional connectivity between the right caudate and bilateral ACC, with this reduction linked to greater cognitive control impairments, such as more errors on the Stroop task. These findings suggest altered right-lateralized frontostriatal dynamics in nicotine dependence (Feng et al., 2016).

Ghosh et al. (2019) examined the integrity of white matter in the orbitofrontal circuit, ACC, inferior frontal circuit, and genu of the corpus callosum among 30 actively opioid-dependent individuals, 30 of their non-substance-dependent brothers, 15 opioid-dependent individuals who had been abstinent for at least one year, and 15 unrelated non-substance-dependent controls. All participants were male and right-handed. Actively opioid-dependent individuals showed lower FA in the bilateral inferior frontal circuit and the right orbitofrontal circuit. Notably, FA reductions in the left inferior frontal circuit were also present in siblings and abstinent individuals, suggesting possible trait markers. The FA in the left and right inferior frontal gyrus was lowest among actively using participants. At the same time, controls demonstrated higher FA in the right OFC compared to the opioid-dependent individuals. No differences in FA were noted for the other regions (Ghosh et al., 2019). These lateralized white matter disruptions may relate to both vulnerability and persistence of opioid dependence.

Taken together, the reviewed studies generally indicate altered hemispheric asymmetries in structural and functional reward networks, often involving left frontostriatal systems in individuals with SUD. While many studies report increased left-hemispheric activation or structural changes compared to controls, some findings are inconsistent or absent (e.g., Sullivan et al., 2005). The direction of alterations frequently suggests left-lateralized dysregulation in salience, valuation, and decision-making circuits, although variations exist across substances, tasks, and samples. These lateralized changes are commonly associated with craving, maladaptive decision-making, and cognitive control impairments, highlighting their potential role in the neuropathology and recovery of SUD.

#### 3.3.2. Cognitive control and memory

SUD is characterized by impairments in cognitive control, working memory, and decision-making, which are crucial for regulating drug-related behaviors (Volkow & Blanco, 2023; Volkow & Boyle, 2018). These cognitive functions are subserved by lateralized brain structures, including the dlPFC, ACC, and hippocampus (Nemati et al., 2023; Roesmann et al., 2019; Wang et al., 2013; Yan et al., 2009). Altered asymmetry in these regions may be associated with executive dysfunction in SUD. The studies below investigate hemispheric asymmetries in neural systems supporting cognitive control and memory in substance-using populations. Table 2 summarizes these studies.

Bell et al. (2014) investigated cortical activations in the response inhibition circuit (RIC) of abstinent cocaine-dependent individuals (3 women, 24 men) compared to age-matched non-using controls (10 women, 35 men) using fMRI during a Go/No-Go motor response inhibition task. The RIC is a critical neural network involved in executive functions such as inhibitory control, frequently impaired in individuals with SUD. This network typically includes regions such as the right middle and inferior frontal gyri, right inferior parietal lobule, bilateral insula, and the midline cingulate and pre-supplementary motor area (pre-SMA). Contrary to studies of active users, no group differences emerged in RIC activation. However, regression analyses indicated that greater activation of the right insula correlated with longer duration of abstinence and higher response success in the cocaine-dependent group (Bell et al., 2014). This suggests a potential adaptive involvement of right-lateralized circuitry during recovery, but inferences are limited by study design.

Kübler et al. (2005) assessed attention switching in 14 cocaine users (6 women, 8 men) and 14 healthy age-matched controls (11 women, 3 men) using fMRI to examine verbal and visuospatial working memory tasks. Participants performed tasks requiring them to update and report on items in either modality or in both simultaneously. Cocaine users displayed significantly poorer performance in the visuospatial task, operating at chance levels, while their performance in the verbal task was comparable to that of controls when comparing the worst and best performers. fMRI results demonstrated significant hypoactivation in several brain regions during attention-switching tasks among cocaine users. On the left side, there was reduced activation in the medial and middle frontal gyri of the PFC, the left cingulate gyrus, the left thalamus, and the lentiform nucleus (globus pallidus/putamen). On the right side, hypoactivation was noted in the right middle frontal gyrus and the right precuneus. This reduced activation was specific to cocaine users and linked to their impaired ability to switch attention effectively. The authors suggested that chronic cocaine use leads to specific rather than generalized deficits in cognitive control, with particular impairment in visuospatial working memory due to disrupted prefrontal and subcortical circuitry (Kübler et al., 2005). Notably, this study shows a high risk of bias according to the NOS; thus, results should be interpreted carefully.

Makris et al. (2008) analyzed cortical thickness asymmetry in the dlPFC using structural MRI in 20 individuals with cocaine dependence and 20 matched controls. Significant group differences in hemispheric asymmetry were observed: while controls exhibited greater right-hemisphere cortical thickness (+2.8 %), individuals with cocaine dependence showed a reversal with greater left-hemisphere thickness (+0.8 %) (Makris et al., 2008). This structural reversal may reflect disrupted lateralized executive network architecture in chronic stimulant use.

Chanraud et al. (2010) explored the impact of dual-tasking on working memory in alcoholics compared to controls, focusing on the frontocerebellar circuitry. The study involved 17 alcohol-dependent subjects and 31 age-matched controls, with more women in the control group compared to the alcohol group. The participants performed verbal and spatial working memory tasks with varying levels of cognitive interference. Both groups showed similar performance on verbal tasks, but alcoholics exhibited a marked deficit in spatial working memory under high-load conditions, particularly when distracted by an arithmetic task. Brain-behavior correlations indicated different neural substrates supporting task performance between groups. In alcohol-dependent subjects, performance was more strongly linked to volumes in the left thalamus and left cerebellar Crus I than in controls, who relied more on the right middle frontal gyrus and right cerebellar Crus I. The authors proposed that alcohol-dependent subjects may utilize different components of the corticocerebellar system, not typically engaged by controls, to compensate for cognitive deficits. This differential engagement may be indicative of altered neural pathways in alcohol-dependent subjects, potentially leading to inefficiencies in handling complex or concurrent tasks. Moreover, a comparison between groups revealed that the alcohol-dependent subjects had smaller volumes in the right superior frontal and the vermian region V1 compared to controls (Chanraud et al., 2010). This suggests altered hemispheric and fronto-cerebellar strategies in alcohol-related executive dysfunction.

Pitel et al. (2013) investigated the functional connectivity between limbic and cerebellar regions in 12 men with alcoholism and 12 matched male controls in an fMRI face-name associative learning task. The study found that alcoholics exhibited lower activation in the left cerebellar Crus II compared to controls, though limbic activation was preserved. Functional connectivity analysis revealed that, at rest, the left hippocampus and left Crus II had positively synchronized activity in controls but were negatively synchronized in alcoholics. Task engagement led to desynchronization in both groups, normalizing the alcoholics’ atypical resting-state synchronization. The authors suggested that alcoholics may have compensatory mechanisms allowing them to perform similarly to controls despite differences in brain activation patterns (Pitel et al., 2013). These findings indicate altered left-lateralized network dynamics potentially compensating for disrupted resting-state connectivity.

De Bellis (2000) measured left and right volumes of the hippocampus in 12 adolescents with alcohol use disorder or addiction (7 females) and 24 matched controls (14 females) (De Bellis, 2000). Individuals with alcohol use disorder or addiction demonstrated smaller left and right hippocampal volumes compared to controls. However, when adjusting for comorbid diagnoses, smaller hippocampal volume was most pronounced in subjects with alcohol use disorder or addiction and comorbid posttraumatic stress disorder (De Bellis, 2000), linking the found structural hemispheric patterns of memory-related neural substrates to SUD severity and comorbidity.

Chumin et al. (2018) investigated the microstructural integrity of white matter in 38 nontreatment-seeking individuals with alcohol dependence (7 women) and 19 social drinkers (3 women), all of whom were cigarette smokers. To achieve this, DTI and structural MRI were performed, and FA images were analyzed using tract-based spatial statistics. Individuals with alcohol dependence exhibited overall lower FA compared to the controls, particularly in the left hemisphere, including the external capsule and the superior longitudinal fasciculus. Additionally, the number of drinks consumed per week was negatively correlated with average FA, irrespective of the group. Finally, tract-based spatial statistics indicated differential connectivity of grey matter in the left frontal, temporal, and parietal regions among SUD subjects compared to the controls (Chumin et al., 2018), suggesting that disrupted left-lateralized white matter integrity is associated with substance use severity.

Yeh et al. (2025) investigated hemispheric asymmetry in individuals with alcohol use disorder (*n* = 25) and healthy controls (*n* = 14) using EEG recordings during virtual reality driving scenarios. Asymmetry indices were calculated from frontal and central electrode pairs using log-transformed power differences between hemispheres. No significant group differences in asymmetry were observed during either resting-state or task performance. These findings suggest that, under both passive and active conditions, alcohol-related alterations in hemispheric activity may not manifest in the EEG measures used or may require more targeted task demands to be detected (Yeh et al., 2025).

Eldreth et al. (2004) investigated brain activation in 25-day abstinent heavy marijuana users and a matched control group of all male participants, using PET during a modified Stroop task. Although task performance did not differ between the groups, significant differences in brain activation were observed. Marijuana users exhibited increased activation in several areas, including the left and right hippocampus, the left occipital lobe (BA18, 19), and the right paracentral lobule (BA6). In contrast, they showed decreased activation in the left perigenual ACC (BA32), the left dlPFC (BA8, 9), the right anterior ventromedial PFC (vmPFC) (BA10), and the right anterior dlPFC (BA10). These region-specific activation differences, while lateralized, may reflect compensatory mechanisms in executive function (Eldreth et al., 2004).

Smith et al. (2013) examined 138 participants, including 42 stimulant-dependent individuals (40 women, 2 men) diagnosed with cocaine (94 %) or amphetamine (6 %) dependence, 49 non-dependent biological siblings (25 women, 24 men), and 47 unrelated healthy controls (17 women, 30 men). They used fMRI while performing the color-word Stroop task to assess cognitive control. Behavioral results revealed significant impairments in cognitive control for both dependent individuals and their siblings, as evidenced by slower response latencies compared to the controls. Neuroimaging indicated that siblings showed notably decreased activation in the inferior frontal gyrus (IFG) and the left superior/middle frontal gyrus compared to both controls and dependent individuals. In contrast, dependent individuals exhibited increased activation in the IFG but did not differ from controls in this regard. The study interpreted these findings as suggesting that both dependent individuals and their siblings possess underlying cognitive inefficiencies, with dependent individuals demonstrating compensatory activation potentially driven by stimulant use. This pattern suggests inherited cognitive control inefficiencies with compensatory lateralized activation in affected individuals (Smith et al., 2013).

Medina et al. (2007) examined right and left hippocampal volumes and hippocampal asymmetry (right-left/right+left) in 16 adolescent alcohol users (five females), 26 alcohol and marijuana users (seven females), and 21 controls (seven females). Alcohol-dependent adolescents showed reduced left hippocampal volume and greater right>left asymmetry compared to both other groups. Interestingly, marijuana abuse/dependence was associated with larger left hippocampal volumes and an increased left>right asymmetry, pointing to substance-specific lateralized effects on hippocampal structure (Medina et al., 2007).

Together, these studies offer correlational evidence of altered hemispheric asymmetry in executive and memory-related circuits in SUD populations. While findings are somewhat variable, there is a general trend toward disrupted lateralization, especially involving the dlPFC and hippocampus. Structural alterations often reflect reduced volume or cortical thickness predominantly in the left hemisphere (e.g., Chumin et al., 2018; Makris et al., 2008; Medina et al., 2007), although some studies report right-lateralized or bilateral effects depending on the substance and task. Functionally, altered activation patterns also display lateralized differences, with some studies indicating increased right-hemispheric recruitment (Bell et al., 2014) during recovery, and others showing compensatory or disrupted engagement of left or right frontal and cerebellar networks (Chanraud et al., 2010; Eldreth et al., 2004). Overall, while the direction and consistency of asymmetry alterations vary across studies and methods, lateralized changes in cognitive control and memory circuits appear to contribute to executive dysfunction and may reflect both vulnerability and compensatory mechanisms in SUD. However, methodological heterogeneity and sample differences necessitate cautious interpretation and underscore the need for future longitudinal and multimodal research to clarify these patterns.

#### 3.3.3. Emotion and salience

Altered emotional processing and dysregulated salience attribution are prominent features of SUD, contributing to heightened cue reactivity, increased stress sensitivity, and elevated risk of relapse (Koob & Volkow, 2016; Volkow & Blanco, 2023). Emotion-related circuits, such as the amygdala, insula, and vmPFC, are functionally lateralized, often showing right hemisphere dominance in emotion processing and interoceptive awareness (Biduła & Króliczak, 2015; Chiarello et al., 2013; Ocklenburg et al., 2022; Reber & Tranel, 2017). Disruptions or shifts in hemispheric asymmetry within these networks may be relevant to affective dysregulation and drug craving in SUD, though these relationships remain correlational rather than causal. This section summarizes findings on lateralized alterations in emotion and salience-related brain systems among substance users. See Table 3 for details of the relevant studies.

Knott et al. (2008) studied 11 regular smokers (5 women) and 11 tobacco chippers (6 women), all of whom were required to abstain from smoking overnight. Participants were first exposed to a control cue (holding a pen in their non-dominant hand), followed by either a depressive or neutral mood induction. Then, they were exposed to a cigarette cue (holding a lit cigarette in their non-dominant hand) to induce craving. EEG resting-state activity was measured during cue exposure, and frequency band asymmetries were calculated. For alpha asymmetry, regular female smokers showed greater left frontal alpha activity during cigarette cue exposure compared to male smokers and light female smokers, suggesting distinct neural responses to cigarette cues in female smokers. No significant effects were found based on the number of cigarettes smoked. During cigarette cue exposure, left frontal theta activity was higher during the depressive mood induction than in the neutral mood condition. Additionally, female participants exhibited greater bihemispheric beta activity in the cigarette cue condition compared to males and the control cue condition. The authors concluded that while smoking cues significantly increased craving, depressed mood, and withdrawal symptoms, the EEG asymmetry results were inconsistent in supporting cigarette craving as a negative state. The observed neural changes in the EEG appeared to be largely independent of the induced depressed mood (Knott et al., 2008). Notably, this study exhibits a high risk of bias according to the NOS; therefore, results should be interpreted with caution.

Faulkner et al. (2020) tested 18 smokers (3 women) and 19 non-smokers (6 women), aged 16 to 21 years, who underwent resting-state fMRI. Whole-brain, voxel-wise connectivity analysis with the bilateral amygdala set as seed region revealed that smoking status modulated the functional connectivity of the bilateral amygdala. Nonsmokers displayed a stronger negative correlation than smokers between emotional clarity scores and the connectivity between the amygdala and the left inferior frontal gyrus. Furthermore, amygdala-to-left inferior frontal gyrus connectivity was significantly weaker in smokers compared to nonsmokers. The authors propose that, considering the inferior frontal gyrus’s role in processing emotional states, enhancing connectivity between the amygdala and the inferior frontal gyrus could potentially improve emotional clarity (Faulkner et al., 2020). Given the role of these regions in emotional processing, such lateralized connectivity differences may reflect altered regulation of negative emotions relevant to smoking behavior.

Deng et al. (2021) examined potential hemispheric asymmetries via EEG in an emotion regulation task preceded by working memory training. Therefore, 40 male (mainly Methamphetamine) abstinent SUD subjects were divided into two groups: one performed a running memory task for 20 days while the other group did not conduct any training (Deng et al., 2021). Before the start of the training and after 20 days, all participants underwent EEG while presented with pictures including neutral, positive, negative, and drug-related stimuli. Then, alpha band power asymmetry scores were calculated for the frontal cortex (mean scores of F3/4, FC3/4, and C3/4) and the post parietal cortex (mean scores of CP3/4, P3/4) during the baseline testing and after training for 20 days. At baseline, asymmetry scores did not differ between groups. Subjects that underwent working memory training showed improved asymmetry scores compared to baseline scores, especially when presented with negative and drug-related stimuli while asymmetry scores declined in controls. Improved left-hemisphere asymmetry after training, especially during negative and drug-related stimuli, suggested enhanced emotion regulation capacity (Deng et al., 2021). Notably, this study shows a high risk of bias according to the NOS; thus, results should be interpreted carefully.

Gilman et al. (2010) conducted a study with 15 alcohol-dependent patients (7 women, 8 men) and 15 age-matched healthy controls (7 women, 8 men). Using fMRI, participants were assessed during three tasks: passive viewing, cognitive judgment (identifying indoor or outdoor scenes), and emotional judgment (expressing liking or disliking of images). The results revealed that alcohol-dependent individuals exhibited greater activation in the left hemisphere, specifically in the inferior frontal gyrus and superior temporal gyrus, as well as in the right middle frontal gyrus, when judging either the location of an image or whether they liked or disliked the image. This increased activation was not observed during passive viewing. The authors suggested that these findings may indicate task-specific lateralization, which reflects compensatory or altered processing strategies (Gilman et al., 2010).

Gizewski et al. (2013) tested 48 male, matched participants, including individuals with schizophrenia (12), alcohol dependence (12), both conditions (12), or neither (12 controls) using fMRI to understand the neural mechanisms underlying cognitive and affective empathy. The participants performed the “Reading the Mind in the Eyes” task, which required them to infer the mental states of individuals based solely on images of their eyes, contrasting this with a gender discrimination control task. The results revealed that participants with schizophrenia showed reduced activation in the left ventrolateral PFC (vlPFC), which was associated with structural deficits in this region, indicating a specific impairment in cognitive empathy. Meanwhile, those with AD exhibited dysfunction in the right anterior insular cortex (AIC), impacting affective empathy. Notably, an interaction effect between schizophrenia and AD was observed, where AD exacerbated deficits in non-schizophrenic individuals but had a different impact on those with schizophrenia. Authors suggested that these findings highlight the role of lateralized brain regions in empathy and suggest that schizophrenia and AD affect different aspects of social cognition. The left vlPFC appears crucial for cognitive empathy, while the right AIC is involved in affective empathy, with their dysfunctions linked to the respective conditions (Gizewski et al., 2013).

The study by De Bellis (2000), described in the section Cognitive Control and Memory also measured left and right volumes of the amygdala in the same adolescents with alcohol use disorder or addiction and matched controls (De Bellis, 2000). However, right and left amygdala volumes did not differ between groups (De Bellis, 2000).

Jung et al. (2007) analyzed structural and surface shape asymmetry of the insula in 20 alcohol-dependent subjects and 20 controls. In patients, a decrease in grey matter and white matter and an increase in cerebrospinal fluid was evident compared to controls. Structural and surface shape analysis revealed distinct deformation patterns in the left and the right insula, resulting in reduced left-right asymmetry, in alcohol-dependent subjects compared to controls (Jung et al., 2007). These reported reduced left-right insular asymmetries in alcohol dependence are consistent with altered lateralized salience processing.

Chumachenko et al. (2015) analyzed differences in cortical thickness in 25 male adolescents with SUD (84 % having alcohol and cannabis abuse or dependence) and 19 male adolescent controls using structural MRI scanning. The authors calculated the mean cortical thickness for the left and right hemispheres separately for the inferior frontal gyrus, orbitofrontal cortex, and insula. Based on these results, they computed the left-right asymmetry for each region. The only significant difference between SUD and control adolescents was a greater right-than-left cortical thickness asymmetry in the inferior frontal gyrus, which was evident in control adolescents but absent in those with SUD. No other analysis, after controlling for age and IQ, revealed significant differences (Chumachenko et al., 2015).

Taken together, these studies provide correlational evidence of altered hemispheric asymmetries across key emotion and salience regions, especially involving the amygdala, insula, inferior frontal gyrus, and vmPFC. Several studies indicate reduced left-right asymmetry or shifts toward right-hemisphere dominance in functional or structural measures (e.g., Jung et al., 2007; Knott et al., 2008), whereas others report disrupted connectivity or compensatory increases in left hemisphere engagement (Faulkner et al., 2020; Gilman et al., 2010). The inconsistencies across findings likely reflect differences in substances, tasks, stages of use or abstinence, and sample characteristics. Overall, these lateralized alterations may contribute to dysregulated emotional processing, impaired salience attribution, and altered interoceptive awareness in SUD, but causal inferences remain limited. Future longitudinal and multimodal studies with larger samples are needed to clarify the directionality, specificity, and clinical implications of these asymmetry changes.

#### 3.3.4. Visual and sensorimotor processing

Although not central to the core symptoms of addiction, visual and sensorimotor systems play an important role in the development of drug-related habits and cue-triggered responses (Yalachkov et al., 2010). These systems are known to exhibit structural and functional lateralization, particularly with visuospatial attention commonly lateralized to the right hemisphere (Nettekoven & Diedrichsen, 2025; Ocklenburg et al., 2024; Vingerhoets, 2019). Alterations in hemispheric asymmetry in these domains may be associated with biased visual attention toward drug cues or changes in habitual motor patterns, although the exact nature and causal direction of these relationships remain to be established. This section reviews studies grouped by neuroimaging modality and behavioral assessment to provide a layered understanding of asymmetry alterations in visual and sensorimotor processing in SUD. Table 4 provides a summary of these studies.

The section begins with structural imaging studies that assess white matter integrity, followed by functional EEG and fMRI investigations, and concludes with behavioral measures of lateralized attention.

Schulte et al. (2010) investigated the impact of white matter fiber degradation on hemispheric asymmetry during visuomotor integration in 17 alcoholics and 16 matched controls, all male, using a combination of DTI and fMRI. Participants performed visual tasks under varying stimulation conditions, including bilateral and unilateral setups. Behavioral results showed that alcoholics exhibited attenuated hemispheric asymmetry, shifting toward more bilateral processing advantages, particularly for right-hand responses. DTI tractography revealed compromised integrity in callosal fibers connecting prefrontal, frontal, and parietal cortices and in left-hemispheric posterior cingulate fibers and left putamen among alcoholics. Functional MRI results indicated reduced activation in the extrastriate cortices of alcoholics, contrasted by preserved thalamic activation and increased cerebellar activation compared to controls. These findings suggest that alcohol-related white matter degradation may contribute to disrupted hemispheric processing asymmetry, which may underlie altered visuomotor integration in affected individuals (Schulte et al., 2010).

Building on these structural findings, Korucuoglu et al. (2016) examined the impact of acute alcohol consumption on motor-related EEG asymmetries during approach-avoidance responses to alcohol cues in 15 heavy-drinking young adults (8 women) and 18 light drinkers (12 women). Alcohol use was assessed using the Alcohol Use Disorder Identification Test. Participants were given either alcoholic beverages or a placebo in separate sessions, and they completed an EEG version of the Alcohol Approach-Avoidance Task. In this task, they were presented with alcohol or soft drink images in different orientations, with instructions to either approach or avoid the images based on orientation. The results showed that light drinkers exhibited positive beta amplitude asymmetries, indicating avoidance-related lateralization for both alcohol and soft-drink cues. In contrast, heavy drinkers showed negative beta amplitude asymmetries, suggesting approach-related lateralization for both cue types. However, in the alcohol condition, this pattern reversed. Heavy drinkers demonstrated greater approach-related lateralization for soft-drink cues, particularly during the late preparation period, indicating an increased asymmetry index in the opposite direction of the expected response. Similar trends were observed for mu- and alpha-related amplitude asymmetries, with higher lateralization of the event-related desynchronization for soft-drink cues in heavy drinkers (Korucuoglu et al., 2016). These results highlight functional asymmetry alterations in sensorimotor processing linked to alcohol use, which may relate to underlying structural changes observed by Schulte et al. (2010).

Rogers et al. (2012) analyzed hemispheric differences in ten right-handed chronic alcohol-dependent patients (5 men, 5 women) and ten matched healthy controls (5 men, 5 women) using fMRI during a finger-tapping task. The task required finger tapping in response to visual cues, and the study focused on functional connectivity between various cortical seed regions and cerebellar targets. The results revealed reduced connectivity specifically between the right PFC (Brodmann Area 9) and bilateral Lobule VIII in the inferior cerebellum, as well as between the right premotor cortex (Brodmann Area 6) and bilateral Lobule VI in the superior cerebellum, in alcohol-dependent patients compared to controls. These connectivity deficits were exclusive to fronto-cerebellar circuits and did not affect other brain regions (Rogers et al., 2012). This fronto-cerebellar dysconnectivity aligns with observed sensorimotor asymmetry alterations and suggests neurobiological damage related to chronic alcohol use that may impact motor functioning.

Finally, behavioral studies provide complementary evidence of altered hemispheric lateralization in visual attention among substance users. Herzig et al. (2010) investigated visuospatial attention in 20 right-handed male smokers and 20 right-handed male non-smokers with a lateralized lexical decision task (reflecting left hemisphere dominance for language) and a lateralized facial decision task (reflecting right hemisphere dominance for visual face recognition). In the lateralized lexical decision task, words were presented on the screen in the combinations: word left/non-word right, non-word left/word right, and non-word/ non-word. In the lateralized facial decision task, 20 sexually dimorphic composite faces and their mirror-reversed appearance with an equal amount of female and male half-faces appearing in each visual field were presented. Participants then had to indicate whether the face seemed female or male. Additionally, participants were asked to fill out the Fagerström Test of Nicotine Dependence and the O-LIFE questionnaire to assess symptoms of schizotypy. Smokers and non-smokers did show the expected lateralization depending on the test: a right visual field advantage in the lateralized lexical decision task and a left-field bias in the lateralized facial decision task. Scores of schizotypy did not affect lateralization but increasing nicotine dependence seemed to predict a right hemisphere bias in both tests (Herzig et al., 2010). These behavioral findings suggest that substance use may subtly shift lateralized visual processing, potentially influencing cue reactivity and addiction behaviors.

In summary, converging evidence from structural, functional, and behavioral studies indicates that substance use is associated with altered hemispheric asymmetries in visual and sensorimotor processing systems. White matter degradation and fronto-cerebellar dysconnectivity in chronic alcohol use appear to disrupt typical lateralized visuomotor integration and motor preparation (Rogers et al., 2012; Schulte et al., 2010). Functionally, heavy drinkers demonstrate atypical EEG asymmetries during approach-avoidance responses, suggesting altered sensorimotor lateralization linked to substance-related cue processing (Korucuoglu et al., 2016). Behavioral findings complement these neuroimaging results, showing subtle shifts in lateralized visual attention correlated with nicotine dependence severity (Herzig et al., 2010). Although the exact causal mechanisms remain unresolved, these alterations may contribute to biased attentional and habitual motor responses that sustain addictive behaviors. Future multimodal and longitudinal research is warranted to elucidate the temporal dynamics and specificity of these lateralized changes in addiction.

#### 3.3.5. Global connectivity

While previous sections focused on domain-specific alterations (e.g., reward processing, emotion, and salience), this brief final section includes studies that examined large-scale or more generalized changes in brain asymmetry that did not align clearly across multiple functional domains. Altered hemispheric asymmetry in large-scale brain networks, spanning multiple functional domains, may reflect correlational evidence of more generalized disruptions in lateralized brain function and connectivity patterns in SUD. This final section, therefore reviews studies examining asymmetry at the level of whole-brain network connectivity in individuals with SUD. Table 5 presents a detailed overview of these studies.

Roemer et al. (1995) tested EEG-based resting-state asymmetry in 90 subjects (35 women) recovering from polysubstance abuse (median = 90 days abstinent) who primarily used cocaine but also reported alcohol and marijuana use. To this end, eyes-closed resting EEG was recorded, and asymmetry of absolute and relative power was calculated in the delta (1.5–3.5 Hz), theta (3.5–7.5 Hz), alpha (7.5–12.5 Hz), and beta (12.5–25 Hz) frequency bands. The authors included details on the duration and amount consumed weekly for all three substances. Interestingly, subjects with increased exposure to cocaine demonstrated reduced right anterior delta power, diminished occipital power in the beta band, frontal interhemispheric asymmetry in the alpha and beta bands, and central hypocoherence in the delta band. Increased alcohol exposure was mainly associated with frontally and temporally reduced delta power, lowered frontal beta power, and a greater left than right hemisphere theta power asymmetry. In contrast, participants with increased marijuana use displayed a greater left power asymmetry in the beta band compared to the right hemisphere and occipital hyper-coherence in the beta band (Roemer et al., 1995). Notably, this study exhibits a high risk of bias according to the NOS; therefore, results should be interpreted with caution.

Hayden et al. (2006) investigated asymmetries in frontal alpha band power among 193 alcohol-dependent subjects (144 men) and 108 control subjects (56 men) during resting state EEG recordings with open and closed eyes. Neither the eye condition (open or closed) nor gender influenced the results; however, when comparing asymmetry scores between the exposure groups, subjects with addiction showed lower left relative to right activation in frontal regions, but not in posterior regions. A comorbid diagnosis of major depression was linked to even less asymmetry in anterior regions (Hayden et al., 2006). These findings suggest an association between alcohol dependence and altered frontal hemispheric activity, but causal relationships cannot be established.

Harris et al. (2008) analyzed white matter integrity in 15 male abstinent long-term chronic alcohol users and 15 control men (Harris et al., 2008). First, a voxel-based analysis was performed to identify regions with differences in FA above a significance threshold to define regions of interest for further analyses. This revealed the orbitofrontal cortex, the cingulum bundle, and superior longitudinal fascicles II and III. Subjects with alcohol use exhibited reduced frontal lobe white matter FA in the right superior longitudinal fascicles II and III, the right orbitofrontal cortex, and the right cingulum bundle compared to controls. No difference was found in the left hemisphere. These right-lateralized structural alterations may relate to disrupted connectivity in networks involved in executive control and reward processing.

Zhu et al. (2018) analyzed global and voxel-wise grey matter asymmetry in 19 alcohol-dependent men and 20 male controls. Global grey matter asymmetry did not differ between groups, but the analysis of voxel-wise grey matter asymmetry revealed different distribution patterns of regions with right- and leftward asymmetry between groups. Especially pronounced was an increased rightward asymmetry of grey matter in the cerebellum (lobules I–IV and V) and lingual gyrus in alcohol-dependent subjects, indicating a shift toward the right hemisphere (Zhu et al., 2018). These structural asymmetry differences align with potential functional alterations in motor and visual networks.

Butcher et al. (2022) examined cerebral blood flow asymmetries in the insular cortex using pseudocontinuous arterial spin labeling MRI in 15 individuals with alcohol use disorder and 22 healthy controls. Compared to controls, the alcohol group exhibited reduced cerebral blood flow bilaterally in the dorsal AIC, as well as reduced perfusion specifically in the left ventral AIC and left posterior insular cortex. While the study did not explicitly quantify hemispheric asymmetry indices, the pattern of left-dominant reductions suggests lateralized alterations in perfusion affecting large-scale salience-related circuits (Butcher et al., 2022). These findings provide preliminary evidence of altered lateralized network function in SUD.

Together, studies investigating global brain connectivity and asymmetry in SUD reveal widespread, lateralized alterations in both structural and functional networks. Resting-state EEG findings indicate substance-specific shifts in power and coherence across multiple frequency bands, reflecting distinct hemispheric disruptions related to cocaine, alcohol, and marijuana use (Roemer et al., 1995). Consistently, alcohol dependence is associated with reduced left relative to right frontal activation and right-lateralized white matter deficits in key tracts implicated in executive control and reward processing (Hayden et al., 2006; Roemer et al., 1995). Voxel-wise analyses further identify shifts toward rightward grey matter asymmetry in motor and visual regions, such as the cerebellum and lingual gyrus (Harris et al., 2008; Zhu et al., 2018). Additionally, lateralized cerebral blood flow reductions within insular subregions suggest altered perfusion in salience-related circuits (Butcher et al., 2022). These findings support the notion that SUD is linked to broad disruptions in hemispheric connectivity and organization, though causal interpretations remain limited. Future research employing longitudinal, multimodal imaging and network-level analyses is needed to clarify the temporal evolution and functional consequences of these lateralized brain alterations in addiction.

### 3.4. Behavioral markers of hemispheric asymmetries

Several behavioral markers of hemispheric asymmetries, including handedness, footedness, markers of visuospatial attention and visual perceptual asymmetries, and asymmetries for acoustic stimuli (Ocklenburg & Güntürkün, 2024), show alterations in several psychiatric conditions (Mundorf & Ocklenburg, 2021). The section will begin with findings on eye dominance, followed by results concerning hemispheric asymmetries in response to acoustic stimuli. Next, studies examining handedness and footedness will be presented. An overview is provided in Table 6.

#### 3.4.1. Eyedness and behavioral markers of visuospatial attention and visual perceptual asymmetries

Differences in eye preference can be examined with several established tests (Ocklenburg & Güntürkün, 2024). Besides questionnaires on eye preference for specific tasks, some studies use a ‘hole-in-the-card-test’ where the subjects have to focus on an object through the hole of a card or paper to discover the preferred eye (Gur, 1977). Then, participants are asked to move the card closer to their face. Meanwhile, the experimenter analyzes to which eye the participant moves the card to, or which eye is closed, thereby establishing which eye shows dominance.

Mandal (2000) tested eye preference, assessed with a side-bias questionnaire (Mandal et al., 1992a), in 30 male heroin-, 30 male alcohol-addicted subjects, and 30 male controls. Subjects were presented with 5 questions (looking through a telescope, snapping a photograph, gun-shooting, looking through a keyhole, preferred eye to wink). They had to indicate their preference on a scale from 1 (never) to 5 (always) for each side. Then, means were calculated and compared between groups. Results confirm a clear right eye preference in controls (mean right: 4.34, mean left: 3.00) and heroin-addicted subjects (mean right: 4.40, mean left: 2.53), but no side bias was found in alcohol-addicted subjects (mean right: 3.61, mean left: 2.90) (Mandal, 2000).

Weinland et al. (2019) analyzed a link between alcohol dependency and eye preference in 200 early-abstinent alcohol-dependent inpatients (113 males, 87 females) and 240 control subjects (133 males, 107 females) by applying a hole-in-the-card test. Alcohol-dependent patients did not differ in eyedness from controls with 47 alcohol-dependent males showing a left eye preference and 55 a right eye preference; 39 alcohol-dependent females a left eye preference and 35 a right eye preference; 60 control males a left eye preference and 71 right eye preference; 42 control females demonstrating a left eye preference and 64 a right eye preference. However, alcohol-dependent subjects with left-eyedness showed a lower risk for alcohol-related readmission as well as fewer and later readmissions, with effects being driven by males (Weinland et al., 2019).

To conclude, findings on eyedness are mixed, with one study indicating reduced lateralization in alcohol-addicted subjects (Mandal, 2000) and another reporting normal right-eye preferences (Weinland et al., 2019). Notably, Weinland et al. (2019) observed that left-eyedness was linked to a lower risk of alcohol-related readmission, suggesting a potential protective effect, particularly in males. Differences in sample size, methodology, and abstinence status likely contribute to these inconsistent results. Therefore, further research with larger, longitudinal samples is warranted to clarify the role of eyedness and its clinical relevance in addiction.

#### 3.4.2. Hemispheric asymmetries for acoustic stimuli

One common way to examine hemispheric asymmetries for acoustic stimuli is the dichotic listening paradigm, in which the participant is simultaneously presented with two different stimuli to both ears played over headphones. The participant then has to indicate which of the two stimuli they heard, and researchers can establish whether the subject demonstrates an advantage of one ear for processing stimuli (Westerhausen & Kompus, 2018). Different versions of this paradigm exist, with most including language stimuli such as consonant-vowel syllables, assessing language lateralization. Some use non-language stimuli. Assumingly, participants with left-hemispheric dominance show a right-ear advantage in this task (Westerhausen & Kompus, 2018), but, depending on the selected stimulus material, varying experimental parameters, and the conditions of stimulus/response selection, a great intra- and inter-individual variability is observed (Westerhausen, 2024; Westerhausen & Kompus, 2018).

Wasserman et al. (1999) investigated an association between substance use and hemispheric asymmetry of the language system in a prospective study with 87 boys (mean age of 9 years). To this end, the participants performed a dichotic consonant-vowel listening test at baseline and after a 3-year follow-up period. Overall, at both time points, all boys had more correct answers with the right than with the left ear. Interestingly, a reduced right ear accuracy (reflective of a deficit in the left hemisphere) at baseline was predictive of substance use at follow-up (Wasserman et al., 1999).

Drake et al. (1990) tested fifteen male and 10 female alcohol-addicted subjects and 15 male and 10 female controls in a verbal dichotic listening task with word pairs such as ‘pop-top’, and ‘goat-coat’. A musical dichotic listening task was also performed with pairs of 2-s violin melodies. In the verbal listening task, alcohol-addicted subjects showed a greater right-ear advantage in both tests compared to controls, which was especially pronounced in males. In the musical dichotic listening task, both groups had more correct responses for left-ear stimuli, and controls had more correct responses than affected subjects with no overall difference between genders (Drake et al., 1990).

Ellis (1990) analyzed dichotic listening in a verbal and a tone task in 22 abstinent male alcohol-dependent patients and 22 male controls. Both groups demonstrated the expected right ear advantage in the verbal task and a left ear advantage for nonverbal, tone stimuli. When correcting for age, no significant difference was evident (Ellis, 1990).

Hahn et al. (2010) investigated hemispheric asymmetries for acoustic stimuli with a consonant-vowel syllable dichotic listening task in 43 smokers (21 females, 22 males) and 47 non-smokers (27 females, 20 males) (Hahn et al., 2010). They subdivided smokers into light (10 females, 9 males) and heavy (11 females, 13 males) smokers based on the Fagerström Test of Nicotine Dependency. Results revealed that all groups showed a strong right-ear advantage. Moreover, heavy-smoking males had lower right ear response scores in the task (mean: 39.27 ± 5.22) compared to light-smoking males (mean: 46.33 ± 3.72) and non-smoking males (mean: 50.25 ± 5.35) and light-smoking males had higher right ear response scores than heavy-smoking men. In female participants, no difference in right ear response scores was evident based on smoking. In line with that, heavy-smoking men showed lower overall right ear response scores compared to heavy-smoking women (Hahn et al., 2010). When comparing task-based calculated laterality index of smokers and non-smokers subdivided by gender, men who were heavy smokers had a lower laterality index compared to light- and non-smoking men. No such difference was found in females. Comparing heavily smoking women with smoking men, women demonstrated a higher laterality index than men. In contrast, non-smoking women showed a lower laterality index than non-smoking men. The authors concluded that male smokers showed a decreased response rate of their right ear indicative of a less lateralized response pattern compared to all other groups. Moreover, this underlines a gender-specific impairment of the speech-dominant left hemisphere in smokers (Hahn et al., 2010).

Mandal (2000) also included questions on ear preference in their study. The same thirty male alcoholic-addicted and 30 male heroin-addicted subjects together with 30 male controls were asked to fill out a side-bias questionnaire including 5 items for ear preference (Mandal et al., 1992a). Subjects had to indicate their hand preference on a scale from 1 (never) to 5 (always) for each side. Then, means were calculated and compared between groups. Results reveal a right ear bias in controls (mean right: 4.67, mean left: 2.45) and heroin-addicted individuals (mean right: 4.80, mean left: 2.27) but again no side bias in alcohol-addicted subjects (mean right: 3.26, mean left: 3.45) (Mandal, 2000).

In sum, findings on hemispheric asymmetries for acoustic stimuli in SUD are mixed. Some studies report an increased right ear advantage in alcohol-dependent adults and male smokers during verbal dichotic listening tasks, suggesting enhanced left-hemispheric processing (Drake et al., 1990; Hahn et al., 2010). Conversely, other research found a reduced right ear advantage in boys at risk for substance use, indicating potential early deficits in left-hemispheric function (Wasserman et al., 1999). Additionally, Mandal (2000) reported no ear preference bias in alcohol-addicted individuals, contrasting with controls and heroin users. These discrepancies may reflect differences in age, substance type, abstinence status, and methodological variations. Further research is needed to clarify the nature and implications of acoustic hemispheric asymmetries in addiction.

#### 3.4.3. Handedness

Hand preference can be assessed through several established methods, such as the Edinburgh Handedness Inventory (EHI) (Oldfield, 1971), the Annett Handedness Inventory (Annett, 1970), or the Shimizu questionnaire (Shimizu & Endo, 1985), all using several questions regarding tool use to assess the preferred hand. A lateralization quotient (LQ) or index can be calculated based on a derived sum score. Next, handedness categories are defined based on cut-off scores and depending on a two-, or three-categorical classification system with left-vs. right-handers or sometimes right- vs. non-right-handedness for a two-categorial system or left- vs. mixed- vs- right-handedness when a three-categorical classification system is used. For a dichotomous classification system, individuals with negative scores are classified as left-handed and subjects with positive scores as right-handed. For the three-category system, several cut-off scores have been proposed. A recent latent class analysis study underlined mixed-handedness’s most plausible cut-off score: an LQ = – 60 to 60 (Mundorf et al., 2024).

Bakan (1973) examined a potential association between left-handedness and alcohol dependency in a sample of 47 alcohol-dependent men. 15 % reported writing with their left hand while 25 % of the sample were classified as non-right-handers (either left-handed or ambilateral) (Bakan, 1973). Notably, this study shows a high risk of bias according to the NOS and thus, results should be interpreted carefully.

Smith and Chyatte (1983) investigated self-reported hand preference rates for writing in 64 patients (51 men) diagnosed with alcohol dependency and abstinent for over 3 months at the time of the study. 39 % of the sample were left-handed (including ambidextrous subjects). The study also included information on the number of previous relapses and found that left-handers relapsed more frequently than right-handers (mean number of relapses: 2.5 for left-handed patients; 0.9 for right-handed patients) (Smith & Chyatte, 1983). Notably, this study shows a high risk of bias according to the NOS; thus, results should be interpreted carefully.

London (1986) assessed handedness with the EHI in a sample of 235 men and 85 women who were admitted for treatment for dependency on alcohol (85 %), opiates (9 %), or cocaine (3 %). A total of 32 men (14 %) and 10 women (12 %) were classified as left-handed based on an LQ < 0. Moreover, left-handers were more likely to be hospitalized than right-handers with an expected rate of 13 % per month for left-handers and 8 % for right-handers (London, 1986). Notably, this study shows a high risk of bias according to the NOS; thus, results should be interpreted carefully.

McNamara et al. (1994) assessed hand preference with the EHI in 43 recovered alcohol-dependent subjects (19 women) who were sober according to self-report. Handedness rates were compared to 311 healthy controls (253 women) and 70 alcohol drinkers at risk of developing alcohol dependency (31 women). Left-handedness was defined by a laterality score below zero. 14.4 % of control men, 20.0 % of problem-drinking men, and 36.8 % of alcohol-dependent men were left-handed according to the EHI. For women, 13.7 % of the controls, 12.9 % of problem-drinking women, and 31.5 % of alcohol-dependent women were left-handed with significant differences in left-handedness between groups for men and women, separately (McNamara et al., 1994). Notably, this study shows a high risk of bias according to the NOS; thus, results should be interpreted carefully.

Bíro and Novotny (1992) analyzed forty-nine alcohol-dependent patients in a tapping test over 8 s (left- and right-hand separately and both hands synchronously) and measured speed for each hand. Furthermore, participants performed a mirror drawing test with a modified Stroop test to induce perceptual load. Finger tapping was performed before and after load exposure. There was a significant difference in speed between the left and the right hand before the load that disappeared after the psychological load. However, no difference was evident when comparing left-hand performance to left-hand performance and right-hand to right-hand performance before and after load. Of interest, 30.6 % of subjects showed faster tapping with their left hand, 53.1 % with their right hand, and 16.3 % had equally good performance with both hands (Bíro & Novotny, 1992). Notably, this study shows a high risk of bias according to the NOS; thus, results should be interpreted carefully.

Bíro (1998) tested twenty-five heroin-addicted patients (11 women) and 25 controls (12 women) in a finger-tapping task with simultaneous tapping of both hands. Then, the number of taps per hand was analyzed. Participants also performed a tracing task, in which they traced several curved lines with a point, first using the right hand followed by the left hand. The number of places touching the line was measured. Even though all participants indicated being right-handed, controls had equal performance in both hands for both tasks. Heroin-addicted subjects showed stronger task-specific side-biases with decreased values for left-hand performance in the tapping test and increased values on the left hand during the tracing task, indicative of a worse performance with the left hand and thus, greater performance with the right hand (Bíro, 1998).

Mandal (2000) also included questions on hand preference in this study. The same thirty male alcohol-addicted and 30 male heroin-addicted subjects together with 30 male controls were asked to fill out a side-bias questionnaire including 22 items for hand preference (Mandal et al., 1992b). Subjects had to indicate their hand preference on a scale from 1 (never) to 5 (always) for each side. Then, means were calculated and compared between groups. Alcohol-addicted subjects did not show a side preference (mean right: 3.95, mean left: 2.80), but heroin-addicted individuals as well as controls demonstrated clear right-hand preferences (mean heroin right: 4.29, left: 2.22; controls right: 4.34, left: 2.08) (Mandal, 2000).

Sperling (2000) and Sperling et al. (2010) assessed handedness with the 13-item Shimizu questionnaire (Shimizu & Endo, 1985) in the same 250 abstinent alcohol-dependent patients (125 women, 125 men), and 250 gender- and age-matched controls. The authors used a two-categorial system classifying the participants into right- and non-right-handers (summarizing mixed- and left-handers). Interestingly, the percentage of non-right-handedness was significantly higher among alcohol-dependent patients compared to controls (12 % in male, and 10.4 % in female controls), but the effect was mainly driven by male patients (44.0 % non-right-handedness in men compared to 6.4 % in women). Notably, the rate of 44 % is substantially higher compared to estimates of around 18 % in the general population (Papadatou-Pastou et al., 2020). In the study, the authors also examined if different subtypes of the disorder show increased percentages of non-right-handedness and found that individuals classified as Type IV in the Lesch typology, and Type II in the Cloninger classification, demonstrate higher rates of non-right-handedness (77.3 % and 45.4 %, respectively). Both classifications are defined by an early onset of problematic drinking behavior. Patients diagnosed with Lesch Type IV often show prenatal cerebral damage and Type II in the Cloninger classification is mostly diagnosed in males (Sperling, 2000; Sperling et al., 2010).

The previously mentioned study by Weinland et al. (2019) also investigated hand preference. Therefore, the same 200 early-abstinent alcohol-dependent inpatients (113 males, 87 females) and 240 control subjects (133 males, 107 females) used the 13-item Shimizu questionnaire. There was no difference in hand preference (non-right-handedness vs. right-handedness) between the groups with 10/7/85 alcohol-dependent males, 3/6/69 alcohol-dependent females, 8/11/114 male controls, and 4/11/92 female controls being left–/ambidexter/right-handed, respectively. The researcher then analyzed whether a crossed eye/hand preference (e.g., left-eye and right-hand preference) was relevant for patients and found that alcohol-dependent patients with crossed eye/hand laterality had a reduced risk for alcohol-related readmission, fewer readmissions, and later readmissions compared to non-crossed patients (Weinland et al., 2019).

In summary, most studies on handedness in individuals with alcohol dependency indicate higher rates of non-right-handedness or reduced right-hand preference compared to controls (Bakan, 1973; London, 1986; McNamara et al., 1994; Smith & Chyatte, 1983; Sperling, 2000; Sperling et al., 2010). This effect appears more pronounced in males and in certain subtypes of alcohol use disorder characterized by early onset or prenatal cerebral damage. In contrast, heroin-dependent individuals generally do not show altered hand preference rates (Bíro, 1998; Mandal, 2000). However, some studies report no significant differences or mixed results (Mandal, 2000; Weinland et al., 2019). The evidence is limited by small samples and risk of bias, warranting further research to confirm these associations.

#### 3.4.4. Footedness

The study mentioned above by Mandal (2000) also included questions on foot preference. The same thirty male alcohol-addicted and 30 male heroin-addicted subjects together with 30 male controls were asked for their foot preference (Mandal, 2000). Side preference was assessed by asking the participants to fill out a questionnaire spanning five items: kicking a ball, foot extended to get into a bus, foot on which body weight rested in standing posture, stepping over an obstacle, foot extended in long jump (Mandal et al., 1992a). Subjects had to indicate their preference on a scale from 1 (never) to 5 (always) for each side. Then, means were calculated and compared between groups. Results show greater right-ward foot preference in controls (right: mean 4.30; left: mean 2.43) and heroin-addicted (right: mean 4.40; left: mean 2.25) compared to alcoholic-addicted subjects that did not exhibit a clear side-bias (right: mean 3.52; left: mean 3.16) (Mandal, 2000). These findings suggest reduced lateralization of foot preference in alcohol-dependent individuals, paralleling similar patterns observed for hand and eye dominance.

## 4. Discussion

This study systematically reviewed the literature on hemispheric asymmetries in substance use and addiction. Structural imaging indicates asymmetric white and grey matter alterations, particularly reduced left-hemispheric white matter integrity and grey matter volume in frontal and temporal regions, while functional data show a tendency toward compensatory right-hemispheric activation during cue reactivity and cognitive tasks, reinforcing the role of lateralized brain function in addiction. Behavioral findings are mixed: while alcohol dependence is linked to higher rates of non-right-handedness, heroin dependence shows no such association. Additionally, auditory lateralization patterns vary by substance, with male smokers showing left-hemisphere deficits and boys at risk for substance use showing reduced right-ear advantage. Based on the synthesis of the reviewed studies, four main conclusions can be drawn, highlighting the substance-specific nature of hemispheric asymmetries, the distinct functional activation patterns across different addictions, the role of behavioral lateralization markers, and the influence of sex and age on these neural differences.

First, the findings suggest that different substances impact hemispheric asymmetry in distinct but overlapping ways, reflecting their unique effects on brain structure and function. Alcohol dependence, for instance, is strongly associated with left-hemisphere reductions in grey and white matter (Chumin et al., 2018; Medina et al., 2007), which may contribute to deficits in cognitive control, language processing, and decision-making (functions typically lateralized to the left hemisphere). This aligns with theories suggesting alcohol-related neurotoxicity preferentially affects the frontal lobes, leading to impulsivity and impaired executive function (Maharjan et al., 2022). Conversely, cocaine dependence shows rightward asymmetry in the supramarginal gyrus and leftward reductions in the striatum (Barrós-Loscertales et al., 2011), which may be linked to disruptions in reward processing and motor control (Kong et al., 2022; Korponay et al., 2022). The right insula’s role in craving and relapse vulnerability (Bell et al., 2014) suggests that right-hemisphere networks could be a target for addiction interventions such as transcranial magnetic stimulation (TMS). For nicotine dependence, the dynamic shifts in lateralization (Feng et al., 2016; Knott et al., 2008) indicate that craving-related neural mechanisms may differ based on the withdrawal state. The right caudate’s role in craving suggests that dopaminergic pathways involved in habit formation might be lateralized in nicotine addiction, raising questions about whether different treatment strategies (e.g., nicotine replacement therapy vs. cognitive interventions) should consider hemispheric differences. These findings emphasize the need for substance-specific neural models of addiction, as different substances appear to target distinct neural circuits. Future research should explore whether these lateralization changes predate addiction or emerge as a consequence, potentially identifying bio-markers for early intervention.

Second, fMRI studies propose that substance use alters lateralized neural networks involved in craving, cognitive control, and emotion regulation. The left-hemisphere reductions in alcohol dependence (Hayden et al., 2006; Pitel et al., 2013; Schulte et al., 2010) contrast with stimulant-related right insula hypoactivation (Balconi et al., 2014; Costumero et al., 2017, 2018; Kübler et al., 2005; Roemer et al., 1995), suggesting that addiction-related dysfunctions are not uniform across substances. This supports the idea that different classes of drugs dysregulate distinct brain circuits, which could have implications for personalized neuromodulation treatments in addiction. Interestingly, marijuana dependence is associated with persistent bilateral hippocampal activation (Eldreth et al., 2004), raising questions about whether chronic cannabis use alters memory networks differently than other substances. This could explain cognitive difficulties reported in long-term cannabis users (Meier et al., 2022) and suggests that treatment approaches should target memory function and cognitive flexibility. The shifts in lateralization based on the withdrawal state in nicotine dependence (Feng et al., 2016; Knott et al., 2008) suggest that craving is a dynamic, state-dependent process, possibly requiring phase-specific interventions (e.g., left-hemisphere stimulation during withdrawal vs. right-hemisphere stimulation during maintenance). These findings emphasize that functional asymmetries are not static; they may fluctuate with substance use patterns, withdrawal states, and individual differences. Future research should examine whether restoring normal hemispheric function through neuromodulation (e.g., TMS, neurofeed-back) could enhance addiction recovery outcomes (Mundorf & Ocklenburg, 2025).

Third, behavioral asymmetries, such as handedness, eye dominance, and auditory processing, may serve as indirect indicators of hemispheric functional imbalances in SUD. The increased prevalence of non-right-handedness in alcohol dependence (Bakan, 1973; Bíro & Novotny, 1992; McNamara et al., 1994; Smith & Chyatte, 1983; Sperling, 2000) aligns with findings in other neuropsychiatric conditions (e.g., schizophrenia, post-traumatic stress disorder, mood disorders; Abbondanza et al., 2023; Packheiser et al., 2025), suggesting that altered lateralization may reflect broader neurodevelopmental vulnerabilities. The question remains whether these differences make individuals more susceptible to addiction or if substance use itself disrupts lateralization. The lack of significant handedness differences in heroin dependence (Mandal, 2000) suggests that not all substances affect hemispheric dominance in the same way. It is possible that heroin’s primary effects on the opioid system and limbic regions do not strongly interact with lateralized processes, whereas alcohol’s impact on the prefrontal cortex and executive function may be more hemisphere-dependent. Dichotic listening studies show reduced right-ear advantage in male smokers (Hahn et al., 2010), suggesting left-hemisphere auditory processing deficits. Interestingly, reduced right-ear advantage has been linked to impulse control deficits and increased risk for externalizing disorders (Combs, 2002; Manassis et al., 2000), supporting the idea that lateralized cognitive vulnerabilities may contribute to addiction severity. These findings highlight the need for further research into lateralization as a potential risk factor for addiction. If certain lateralization patterns precede substance use, they could serve as early markers for prevention efforts.

Fourth, sex and age differences in SUD-related asymmetries highlight the importance of individualized approaches to addiction treatment. Men with alcohol dependence show higher rates of non-right-handedness (Sperling, 2000) while prevalences of non-right-handedness are solely moderately increased in women (London, 1986; McNamara et al., 1994; Sperling, 2000), suggesting that sex-specific interventions may be beneficial. The left-hemisphere deficits in male smokers (Hahn et al., 2010) and bihemispheric activity in female smokers (Knott et al., 2008) indicate different neural compensation strategies, raising the possibility that men and women may respond differently to smoking cessation therapies. Future studies should explore whether sex-based differences in lateralization predict relapse risk or treatment success. Adolescents with substance use (alcohol, cannabis, stimulants) show greater rightward cortical asymmetry (Chumachenko et al., 2015), supporting the idea that early substance exposure disrupts normal brain development. This could have long-term consequences for cognitive control and risk-taking behavior, reinforcing the need for early intervention strategies. In contrast, older adults with long-term alcohol dependence show white matter disruptions primarily in frontal and parietal networks (Chumin et al., 2018; Harris et al., 2008; Jung et al., 2007). This suggests that chronic alcohol use may exacerbate natural age-related cognitive decline, potentially increasing dementia risk (Roe et al., 2021). Targeted cognitive rehabilitation or neuroprotective treatments could help mitigate these effects. These findings stress the importance of age- and sex-specific treatment approaches tailored to substance-specific neural vulnerabilities. Future research should examine how developmental and hormonal factors shape hemispheric asymmetries in addiction.

Taken together, a one-size-fits-all approach to addiction treatment may be insufficient given the substance-specific, sex-dependent, and age-related differences in brain asymmetries. Understanding how different drugs alter hemispheric function could help refine neuromodulation techniques, cognitive therapies, and pharmacological treatments. Future studies should explore whether restoring normal lateralization patterns can enhance addiction recovery and reduce relapse risk.

## 5. Implications for future research and treatment

These findings emphasize the necessity for sex- and age-specific approaches to understanding addiction-related hemispheric asymmetries. Future studies should examine whether sex-based differences in lateralization impact treatment outcomes (e.g., do left-handed or right-handed men and women respond differently to addiction therapies?). Furthermore, future research must explore how early-life substance exposure influences long-term hemispheric development and whether these changes heighten susceptibility to chronic addiction. Additionally, studies should investigate whether age-related white matter decline in older substance users can be slowed or reversed through targeted interventions. Advancing this work will also require more methodologically rigorous investigations of brain asymmetries. In particular, few studies with adequately powered samples have examined resting-state or task-based asymmetries using established, formal approaches (e.g., lateralization indices), representing a critical gap that should be prioritized in future research.

## 6. Limitations

The review process has certain limitations. Systematic reviews are often influenced by publication bias, where studies that support the research hypothesis are more likely to be published, while those with null results tend to be overlooked. This is especially problematic when the body of evidence is still relatively small, as is the case with laterality and SUD. The inclusion of explicit hemispheric asymmetry investigation as a criterion may lead to the exclusion of studies that could have been overlooked using the current search strategy. Moreover, it is crucial to recognize that changes in asymmetry can arise from various factors, and unilateral alterations may not necessarily lead to altered asymmetries. Consequently, the relationship between unilateral changes and asymmetry alterations remains unclear. Unfortunately, many studies did not report results for both hemispheres or examine any potential connection between them. In terms of findings, most studies focus on alcohol dependence, with limited research on other substances. Findings on heroin users suggest substance-specific effects, as no significant asymmetry changes were found in heroin or nicotine users. Many studies rely on cut-off scores rather than clinical diagnoses, raising concerns about comparability. Age differences also pose a challenge, as studies mix young and older adults despite evidence that asymmetries may change over time (Ford et al., 2023; Roe et al., 2023). Additionally, handedness assessment varies widely, making replication difficult (Edlin et al., 2015; Mundorf et al., 2024; Yeung & Wong, 2022). Similarly, sex differences are often overlooked, even though men and women may show distinct lateralization patterns. The use of different neuroimaging methods (MRI, fMRI, EEG) and analysis techniques complicates cross-study comparisons, while the predominance of cross-sectional studies makes it unclear whether asymmetry changes cause or result from substance use. Psychiatric comorbidities such as depression and anxiety may further influence findings, making it difficult to isolate the effects of SUD. Moreover, polysubstance use is rarely considered, despite being common in real-world addiction. Finally, small sample sizes limit statistical power, highlighting the need for larger, standardized studies to improve reliability and generalizability.

## 7. Conclusion

The review highlights significant alterations in both grey and white matter asymmetries in individuals with SUD. Most findings suggest reduced white matter integrity and decreased grey matter volume, particularly in regions associated with cognitive control, reward processing, and impulse regulation. Functional imaging studies point toward disrupted hemispheric connectivity and lateralized activation patterns, with notable differences in resting-state and task-based brain activity. The results indicate that behavioral asymmetries, such as handedness and ear dominance, may be associated with substance use. Some studies suggest an increased prevalence of non-right-handedness in alcohol-dependent individuals, while others report mixed findings on ear and eye dominance. These findings raise questions about whether altered lateralization is a risk factor for addiction or a consequence of prolonged substance use. While alcohol dependence has been extensively studied, findings on other substances, such as nicotine and heroin, suggest that altered hemispheric asymmetries may vary depending on the type of substance used. Additionally, the findings highlight the need for sex- and age-specific approaches to understanding addiction-related hemispheric asymmetries. Given that different substances uniquely affect lateralization patterns, future research and treatment strategies should consider both substance type and demographic factors. Moreover, differences in study methodologies, including sample demographics, handedness classification, and neuroimaging techniques, contribute to inconsistencies in the literature. Future research should aim for standardized measures to improve comparability across studies.

## Supplementary Material

1

## Figures and Tables

**Table 1:** Reward processing. Summary of studies reporting measures of, or findings related to, reward processing in subjects with substance abuse or addiction (SUD) and controls. Only results concerning hemispheric differences are reported. Symbols indicate directionality of effects: ↑ = increased; ↔ = no left-right difference in the observed alterations; ↓ = reduced (in SUD compared to controls). Asymmetry Assessment refers to the method used to evaluate hemispheric differences. Possible methods include: AI: Asymmetry index was calculated; Stat test: statistical tests comparing hemispheres were used; L/R values only: left and right values were reported without comparison.

Study	Sample (n)	Substance	Mean age in years ± SD	Gender ratio (f/m)	Handedness (LH/RH)	Assessment of SUD	Neuroimaging Method	Asymmetry Assessment	Results
Balconi et al., 2014	SUD: 40 CG: 42	Cocaine	SUD: 55.3 ± 4.33 CG: 56.98 ± 5.09	SUD: 17/23 CG: 18/24	*Not specified*	Met criteria based on SCID	EEG + Iowa Gambling Task	AI = (ln [right] –ln [left])	↑ L hemispheric activation
Costumero et al., 2017	SUD: 20 CG: 21	Cocaine	SUD: 37.2 ± 6.8 CG: 36.1 ± 8	All male	All RH	DSM-IV criteria met	Task-based fMRI Cue-reactivity task	Stat test: ICA Component based GLM activation	SUD: ↓ activity for non-drug rewarding stimuli ↓ L FPN
Barrós-Loscertales et al., 2011	SUD: 20; CG: 16	Cocaine	SUD: 33.30 ± 6.94 CG: 33.38 ± 9.17	All males	All RH	Met DSM-IV criteria	1.5 T MRI, GM volume	Stat test: Whole brain voxel-wise analyses	↓ GM L striatum (14.8 %), ↓ GM R supramarginal gyrus (12.1 %)
Tapert et al., 2003	SUD: 15 CG:15	Alcohol	SUD: 16.96 ± 0.78 CG: 16.35 ± 1.02	SUD: 6/9 CG: 6/9	All RH	DSM-IV criteria met	Task-based fMRI Cue reactivity task	Stat test: Voxel-wise GLM activation	↓ R middle frontal gyrus ↓ R inferior frontal gyrus ↑ L medial frontal and paracentral gyri ↑ L dorsal cingulate and paracentral gyri ↑ L prefrontal and orbital gyri ↑ L superior and middle frontal gyri ↑ bilateral inferior frontal gyrus ↑ L ventral anterior cingulate and subcallosal cortex ↑ bilateral parahippocampus and amygdala ↑ R uncus ↑ L middle to inferior temporal and fusiform gyri ↑ L middle to superior temporal gyri ↑ L hypothalamus ↑ bilateral posterior cingulate and precuneus ↑ bilateral cuneus ↑ L angular gyrus ↑ R precuneus and lateral precuneus ↑ R lateral precuneus
Beylergil et al., 2017	SUD: 34 CG: 26	Alcohol	SUD: 44.73 ± 8.27 CG: 41.92 ± 9.59	All male	All RH	ADS + OCDS + LDH	Task-based fMRI (Reward-guided decision-making task)	Stat test: Voxel-wise GLM activation	reduced PE-related activity in bilateral dlPFC ↓ L dlPFC - negative PE ↓ R dlPFC - positive PE
Sullivan et al., 2005	SUD: 25; CG: 51	Alcohol	SUD: 49.4 ± 10.9 CG: 45.2 ± 13.9	All males	*Not specified*	Inpatient, met DSM-III-R criteria	1.5 T MRI ROI volumes	Stat test: Left vs right	↔ volume in caudate nucleus, putamen, nucleus accumbens, and medial septal/diagonal band
Feng et al., 2016	SUD: 27 CG: 25	Alcohol	SUD: 20.7 ± 1.5 CG: 20.5 ± 1.4	All male	All RH	DSM-V criteria + FTND; consuming ≥10 cigarettes/day in last 6 mos	Resting-state fMRI	Stat test: Seed-based GLM connectivity	↓ R caudate - bilat. ACC ↓ R caudate - left hippocampus ↑ R caudate – correlated with craving
Ghosh et al., 2019	SUD: 30; Abstinent SUD: 15; CG brothers: 30; CG: 15	Opioids	SUD: 27.8 ± 4.2; Abstinent SUD: 30.1 ± 5.5; CG brothers: 28.4 ± 6.3; CG: 33 ± 5.5	All males	All RH	ICD-10 + DSM-IV-TR criteria; clinical interview	30-direction DTI, WM integrity	L/R values only	SUD: ↓ FA bilateral inferior frontal circuit; abstinent SUD + CG brothers: ↓ FA L inferior frontal circuit; ↔ other frontal regions CG: ↑ FA R OFC

ACC Anterior cingulate cortex.

ADS Alcohol Dependent Scale.

AI Asymmetry Index.

CG Control group.

dlPFC Dorsolateral Prefrontal Cortex.

DTI Diffusion Tensor Imaging.

FA Fractional anisotropy.

FC Functional connectivity.

fMRI Functional magnetic resonance imaging.

FTND Fagerström Test of Nicotine Dependence.

FPN Fronto-parietal network.

GM Grey matter.

L Left.

LDH Lifetime Drinking History questionnaire.

LH Left-handers.

MRI Magnetic resonance imaging.

OCDS Obsessive Compulsive Drinking Scale.

OFC Orbitofrontal cortex.

PE Prediction error.

PFC Prefrontal Cortex.

SCID Structured Clinical Interview for DSM-IV Axis I Disorder.

R Right.

RH Right-handers.

WM White matter.

VBM Voxel-based morphometry.

**Table 2:** Cognitive control and memory. Summary of studies examining hemispheric asymmetries in brain regions related to cognitive control and memory in individuals with SUD compared to controls. Included studies assessed structures such as the prefrontal cortex, and hippocampus. Results are limited to those reporting left–right differences. Symbols indicate directionality of effects: ↑ = increased; ↔ = no left-right difference in the observed alterations; ↓ = reduced (in SUD compared to controls). Asymmetry Assessment refers to the method used to evaluate hemispheric differences. Possible methods include: AI: Asymmetry index was calculated; Stat test: statistical tests comparing hemispheres were used; L/R values only: left and right values were reported without comparison.

Study	Sample (n)	Substance	Mean age in years ± SD	Gender ratio (f/m)	Handedness (LH/RH)	Assessment of SUD	Neuroimaging Method	Asymmetry Assessment	Results
Bell et al., 2014	SUD: 27 CG: 45	Cocaine	SUD: 37.8 ± 7.8 CG: 38.1 ± 10.6	SUD:3/24 CG:10/35	*Not specified*	SCID for DSM-IV diagnosis, urine toxicology tests	Task-based fMRI (Go/No-Go motor response inhibition task)	Stat test: Voxel-wise GLM activation	No difference in RIC ↑ R anterior insula - ↑ task success and ↑ abstinence duration
Kübler et al., 2005	SUD: 14 CG: 14	Cocaine	SUD: 24.3 ± 3.8 CG: 37.6 ± 6.4	SUD:11/3 CG: 6/8	All RH	Drug history, min 2 years + Urine samples	Task-based fMRI Verbal and visuospatial working memory task	Stat test: Voxel-wise GLM activation	↓ L cingulate gyrus ↓ R middle frontal gyrus ↓ L thalamus ↓ R precuneus
Makris et al., 2008	SUD: 20; CG: 20	Cocaine	SUD: 34.3 ± 10.8 CG: 34.1 ± 9.4	SUD: 11/9 CG: 11/9	SUD: 4/16 CG: 4/16	SCID + ASAM-PPC2R; SCID criteria met	1.5 T MRI, thickness	AI = L _volume_ – R _volume_ / 1/2 (L _volume_ + R _volume_)	SUD: L > R dlPFC thickness (+0.8 %) CG: R > L dlPFC thickness (+2.8 %)
Chanraud et al., 2010	SUD: 17 CG: 31	Alcohol	SUD: 44.0 ± 10.3 CG: 40.4 ± 12.1	more women in the control than the alcoholic group	All RH, determined with the Crovitz Handedness Questionnaire	SCID, DSM-IV criteria met	Task-based fMRI Verbal and spatial working memory Interference tasks: arithmetic and tracking Structural	L/R values only	↔ in ROIs explored but structure–function correlations were observed ↔ L precentral ↔ L thalamus ↔ L cerebellar Crus I ↓ volumes in R superior frontal and vermian region V1
Pitel et al., 2013	SUD: 12 CG:12	Alcohol	SUD: 38.0 ± 7.9 CG: 43.0 ± 11.0	All male	All RH	DSM-IV by clinical research psychologists or research nurse	Task-based fMRI Face-name association memory test	Stat test: Voxel-wise GLM activation	↓ L cereballar Crus II connectivity at rest; ↓ L hippocampus - L Crus II
De Bellis, 2000 *	SUD: 12 CG: 24	Alcohol	SUD: 17.2 ± 2.2 CG: 17.0 ± 2.4	SUD: 7/5 CG: 14/10	Matched but not specified	SCID, interviewer defined diagnoses	1.5 T MRI, GM + WM	L/R values only	↓ bilateral hippocampus
Chumin et al., 2018	SUD: 38; CG: 19	Alcohol	SUD: 38.6 ± 8.1; CG: 37.8 ± 8.6	SUD: 7/31; CG: 3/16	AS: 0/2(A)/36 CG: 0/19	SSAGA + ADS; DSM-IV criteria met	3.0 T DTI, structural MRI	Stat test: Voxel-wise analysis	↓ FA in L external capsule + superior longitudinal fasciculus
Yeh et al., 2025	SUD: 25 CG: 14	Alcohol	SUD: 43.75 ± 7.89 CG: 36.5 ± 10.57	SUD: 5/20 CG: 9/5	*Not specified*	AUDIT, Diagnosed by psychiatrist	EEG + Virtual Reality Driving scenarios	AI = (ln [right] –ln [left])	No differences in AI
Eldreth et al., 2004	SUD: 11 CG: 11	Marijuana	SUD: 25 (21–35) CG: 29 (22–34)	All male	All RH	DUSQ + ASI + DIS	Task-based PET Stroop task	Stat test: Voxel-wise GLM activation	↓ L perigenual ACC ↓ L dlPFC ↑ bilateral hippocampus ↑ R paracentral lobule ↑ L occipital lobe ↓ R anterior vmPFC ↓ R anterior dlPFC
Smith et al., 2013	SUD: 42 CG: 47	Cocaine Amphetamine Alcohol	SUD:34.24 ± 7.39 CG: 32.34 ± 8.63	SUD: 2/40 CG: 17/30	*Not specified*	DSM-IV + OCDUS + AUDIT	Task-based fMRI Color-word Stroop task	Stat test: Voxel-wise GLM activation	Siblings vs SUD & CG ↓ L inferior frontal gyrus ↓ L superior frontal gyrus ↓ L middle frontal gyrus
Medina et al., 2007	SUD(1): 16 SUD(2): 26 CG: 21	Alcohol (1), Alcohol + Marijuana (2)	SUD(1): 16.9 ± 0.7 SUD(2): 17.6 ± 0.9 CG: 17.5 ± 1.1	SUD(1): 5/11 SUD(2): 7/19 CG: 7/14	All RH	C-DIS-IV, screening interviews to assess eligibility	1.5 T MRI, ROI	AI = (RH - LH/RHr + LH)	↓ L volume + ↑ right>left asymmetry in hippocampus

ACC Anterior cingulate cortex.

ADS Alcohol Dependent Scale.

AI Asymmetry Index.

ASAM-PPC2R American Society of Addiction Medicine Patient Placement Criteria 2-Revised.

AUDIT Alcohol Use Disorders Identification Test.

C-DIS-IV Computerized Diagnostic Interview Schedule.

CG Control group.

DCQ Drug Craving Questionnaire.

DIS Diagnostic Interview Schedule.

DUSQ Drug Use Survey Questionnaire.

dlPFC Dorsolateral Prefrontal Cortex.

DTI Diffusion Tensor Imaging.

FA Fractional anisotropy.

FTND Fagerström Test of Nicotine Dependence.

FC Functional connectivity.

fMRI Functional magnetic resonance imaging.

GM Grey matter.

L Left.

LH Left-handers.

MRI Magnetic resonance imaging.

OCDUS Obsessive Compulsive Drug Use Scale.

OFC Orbitofrontal cortex.

PFC Prefrontal Cortex.

SCID Structured Clinical Interview for DSM-IV Axis I Disorder.

SCID-P Structured Clinical Interview for DSM-IV Axis I Disorder, Patient Edition.

SSAGA Semi-Structured Assessment for the Genetics of Alcoholism.

R Right.

RH Right-handers.

RIC Response inhibition circuit.

WM White matter.

VBM Voxel-based morphometry.

vmPFC Ventromedial Prefrontal Cortex.

vlPFC Ventrolateral Prefrontal Cortex.

* This study appears in Tables 2 and 3 as results fit to both.

**Table 3:** Emotion and salience. Summary of studies reporting asymmetries in emotion- and salience-related brain regions (e.g., amygdala, insula, ventromedial prefrontal cortex) in individuals with SUD. Only findings concerning hemispheric differences are presented. Symbols indicate directionality of effects: ↑ = increased; ↔ = no left-right difference in the observed alterations; ↓ = reduced (in SUD compared to controls). Asymmetry Assessment refers to the method used to evaluate hemispheric differences. Possible methods include: AI: Asymmetry index was calculated; Stat test: statistical tests comparing hemispheres were used; L/R values only: left and right values were reported without comparison.

Study	Sample (n)	Substance	Mean age in years ± SD	Gender ratio (f/m)	Handedness (LH/RH)	Assessment of SUD	Neuroimaging Method	Asymmetry Assessment	Results
Knott et al., 2008	SUD: 11 CG: 11	Tobacco	SUD (f): 22.2 ± 1.60 SUD (m):25.8 ± 1.46 CG (f):20.8 ± 1.46 CG (m): 26.2 ± 1.60	SUD: 5/6 CG: 6/5	*Not specified*	FTND, smoking max. of 5 cigarettes/day, at least 2 days/week	EEG resting-state; mood + caring induction	AI = (L – R)/(L + R)	↑ L frontal alpha activity in ♀ smokers during cigarette cue ↑ L frontal theta activity during cigarette cue in depressed mood ↑ Bihemispheric beta activity in ♀ during cigarette cue
Faulkner et al., 2020	SUD: 18 CG: 19	Tobacco	SUD: 19.11 ± 4.6 CG: 19.89 ± 1.2	SUD: 4/14 CG: 6/13	*Not specified*	DERS scale + FTND; daily smoking for >6 months	Resting-state fMRI	Stat test: Seed-based GLM connectivity	↓ amygdala-to-left inferior frontal gyrus connectivity
Deng et al., 2021	SUD + training: 20 SUD + no training: 20	Methamphetamine	SUD+: 36.30 ± 7.97 SUD−: 35.20 ± 6.69	SUD+: 0/20 SUD−: 0/20	All RH	DCQ, undergoing drug abstinence	EEG + Emotional pictures	AI = (ln [right] –ln [left])	↑ L hemispheric activation for negative + drug-related stimuli after training
Gilman et al., 2010	SUD: 15 CG: 15	Alcohol	SUD: 35.2 ± 7.34 CG: 33.3 ± 8.60	SUD: 7/8 CG: 7/8	All RH	DSM-IV criteria met	Task-based fMRI Visual judgment task	Stat test: Voxel-wise GLM activation	↑ L Inferior Frontal Gyrus ↑ L Superior Temporal Gyrus ↑ R Middle Frontal Gyrus
Gizewski et al., 2013	SUD: 12 CG: 12	Alcohol	SUD: 37.0 ± 8.2 CG: 36.6 ± 11.2	All male	All RH	SCID by psychiatrist	Task-based fMRI Mind reading task	Stat test: Voxel-wise GLM activation	L ventrolateral PFC ↓ Right anterior insular cortex (BA13)
De Bellis, 2000 *	SUD: 12 CG: 24	Alcohol	SUD:17.2 ± 2.2 CG: 17.0 ± 2.4	SUD: 7/5 CG: 14/10	Matched but not specified	SCID, interviewer defined diagnoses	1.5 T MRI, GM + WM	L/R values only	↔ bilateral amygdala
Jung et al., 2007	SUD: 20; CG: 20	Alcohol	SUD: 43.5 ± 6.0 CG: 44.5 ± 7.4	*Not specified*	*Not specified*	SCID	3.0 T MRI, ROI structural and surface shape	AI = (|L − R| / |L| + |R|) × 2	↓ L-R insula shape asymmetry
Chumachenko et al., 2015	SUD: 25; CG: 19	*Not specified*	SUD: 16.64 ± 1.15 CG: 16.59 ± 1.62	All males	All RH	DSM-IV criteria met; DISC-IV + CIDI-SAM	3.0 T 3D MRI, cortical thickness	AI = (LCT-RCT)/[0.5*(LCT + RCT)] LCT + RCT = cortical thicknesses of left or right ROI	↓ R > L cortical thickness in inferior frontal gyrus

AI Asymmetry Index.

CG Control group.

CIDI-SAM Composite International Diagnostic Interview - Substance Abuse Module.

DCQ Drug Craving Questionnaire.

DERS Difficulties in Emotion Regulation Scale.

DISC-IV Diagnostic Interview Schedule for Children-Version IV.

DTI Diffusion Tensor Imaging.

FA Fractional anisotropy.

FTND Fagerström Test of Nicotine Dependence.

FC Functional connectivity.

fMRI Functional magnetic resonance imaging.

GM Grey matter.

L Left.

LH Left-handers.

MRI Magnetic resonance imaging.

OFC Orbitofrontal cortex.

PE Prediction error.

PFC Prefrontal Cortex.

SCID Structured Clinical Interview for DSM-IV Axis I Disorder.

SCID-P Structured Clinical Interview for DSM-IV Axis I Disorder, Patient Edition.

SSAGA Semi-Structured Assessment for the Genetics of Alcoholism.

R Right.

RH Right-handers.

WM White matter.

VBM Voxel-based morphometry.

* This study appears in Tables 2 and 3 as results fit to both.

**Table 4:** Visual and sensory-motor-related tasks or regions. Summary of studies examining lateralized alterations in visual and sensorimotor regions in the context of SUD. This includes areas involved in visuospatial attention, perceptual processing, and motor function. Reported findings are restricted to those addressing hemispheric asymmetries. Symbols indicate directionality of effects: ↑ = increased; ↔ = no left-right difference in the observed alterations; ↓ = reduced (in SUD compared to controls). Asymmetry Assessment refers to the method used to evaluate hemispheric differences. Possible methods include: AI: Asymmetry index was calculated; Stat test: statistical tests comparing hemispheres were used.

Study	Sample (n)	Substance	Mean age in years ± SD	Gender ratio (f/m)	Handedness (LH/RH)	Assessment of SUD	Neuroimaging Method	Asymmetry Assessment	Results
Schulte et al., 2010	SUD: 17 CG:16	Alcohol	SUD: 51 ± 7.2 CG: 49 ± 15.3	All male	All RH, except for 2 (1 CG and 1 SUD)	SCID clinical interview	DTI combined with task-based fMRI Visual field testing	Stat test: Voxel-wise GLM activation	↓ L posterior cingulate ↓ L putamen
Korucuoglu et al., 2016	SUD: 15 CG: 18	Alcohol	SUD: 17.4 ± 1.24 CG: 18 ± 1.19	SUD: 8/7 CG: 12/6	All RH	AUDIT >8	Alcohol administration, EEG + Alcohol approach avoidance task	Stat test	↑ L hemispheric activation for avoidance-related stimuli in CG ↑ R hemispheric activation for approach-related stimuli in SUD, particularly for soft-drink cues during late preparation
Rogers et al., 2012	SUD:10 CG: 10	Tobacco	SUD: 43 ± 12 CG: 40 ± 13	SUD: 3/7 CG: 3/7	All RH	DSM-IV criteria, diagnosed by psychiatrist	Task-based FC Connectivity fMRI; finger tapping	Stat test: Seed-based GLM connectivity	↓ R prefront (BA 9) - inf. Cerebellum - R lobule VIII) ↓ R premotor (BA6) - sup. Cerebellum (bilat. Lobule VI)
Herzig et al., 2010	SUD: 20 CG: 20	Nicotine	SUD: 22 ±2 CG: 21 ±1	SUD: 0/20 CG: 0/20	All RH	FTND	Lateralized lexical decision + lateralized facial decision task	Visuospatial attention	↔; Nicotine dependence ↑ R bias in both tasks

AUDIT Alcohol Use Disorders Identification Test.

CG Control group.

DTI Diffusion Tensor Imaging.

FTND Fagerström Test of Nicotine Dependence.

FC Functional connectivity.

fMRI Functional magnetic resonance imaging.

L Left.

LH Left-handers.

SCID Structured Clinical Interview for DSM-IV Axis I Disorder.

R Right.

RH Right-handers.

**Table 5:** Global connectivity. Summary of studies investigating hemispheric asymmetries in large-scale brain networks and global connectivity patterns in individuals with SUD. This includes intra- and inter-hemispheric alterations in connectivity involving default mode, salience, and executive networks. Only findings reporting left–right differences are included. Symbols indicate directionality of effects: ↑ = increased; ↔ = no left-right difference in the observed alterations; ↓ = reduced (in SUD compared to controls). Asymmetry Assessment refers to the method used to evaluate hemispheric differences. Possible methods include: AI: Asymmetry index was calculated; Stat test: statistical tests comparing hemispheres were used; L/R values only: left and right values were reported without comparison.

Study	Sample (n)	Substance	Mean age in years ± SD	Gender ratio (f/m)	Handedness (LH/RH)	Assessment of SUD	Neuroimaging Method	Asymmetry Assessment	Results
Roemer et al., 1995	SUD: 90	Cocaine	*Not specified*	35/55	*Not specified*	SCID-P, met DSM-III-R criteria	Quantitative EEG resting-state	AI, not specified	Cocaine: ↓ R anterior delta power, ↓ Occipital beta power, frontal asymmetry in alpha + beta bands Alcohol: ↓ Frontal and temporal delta power, ↓ Frontal beta power, ↑ L theta power asymmetry Marijuana: ↑ L beta power asymmetry, ↑ Occipital beta hypercoherence
Hayden et al., 2006	SUD: 193; CG: 108	Alcohol	SUD: 43 ± 11 CG: 41 ± 14	SUD: 49/144 CG: 52/56	All RH	SSAGA, DSM-III-R criteria met	EEG resting-state	AI = (ln [right] –ln[left])	↓ L relative to R frontal alpha band power
Harris et al., 2008	SUD: 15; CG: 15	Alcohol	SUD: 48.3 ± 13.1; CG: 56.4 ± 9.0	All males	All RH	medical history interview + DIS; DSM-IV criteria met	3.0 T DTI MRI + VBM	Stat test: right vs. left	↓ FA R superior longitudinal fascicles II and III, R OFC, R cingulum bundle
Zhu et al., 2018	SUD: 19; CG: 20	Alcohol	SUD: 38.7 ± 7.8; CG: 42.9 ± 11.5	All males	All RH	SCID-P, inpatients	3.0 T 3D-MRI, global + voxel-wise GM asymmetry	AI = *i*1 - *i*2 / 0.5 × (*i*1 + *i*2) *i*1 and *i*2 = modulated original + flipped GM images	↔ global GM asymmetry; Differential voxel-wise GM asymmetry pattern; ↑ R asymmetry in cerebellum (lobules I-IV, V) + lingual gyrus
Butcher et al., 2022	SUD: 15 CG: 22	Alcohol	SUD: 36.5 ± 11.2 CG: 35.6 ± 11.9	SUD: 6/9 CG: 13/9	*Not specified*	SSAGA + AUDIT; DSM-IV criteria met	pseudocontinuous arterial spin labeling MRI	L/R values only	↓ CBF L + R dorsal AIC ↓ CBF L ventral AIC ↓ CBF L PIC

AI Asymmetry Index.

AIC Anterior insular cortex.

AUDIT Alcohol Use Disorders Identification Test.

CBF Cerebral Blood Flow.

CG Control group.

DIS Diagnostic Interview Schedule.

EEG Electroencephalogram.

L Left.

LH Light-handers.

MRI Magnetic resonance imaging.

PIC posterior insular cortex.

R Right.

RH Right-handers.

SCID-P Structured Clinical Interview for DSM-IV Axis I Disorder, Patient version.

SSAGA Semi-Structured Assessment for the Genetics of Alcoholism.

**Table 6:** Behavioral lateralization. Summary of studies including measures of behavioral lateralization in subjects with substance abuse or addiction (SUD) and controls. ↑ = increased/stronger, ↓ = reduced in affected compared to controls, ↔ = no difference in affected compared to controls.

Lateralized behavior	Study	Sample (n)	Substance	Mean age in years ± SD	Gender ratio (f/m)	Handedness (LH/RH)	Assessment of SUD	Laterality Assessment	Results
Eyedness	Mandal, 2000	SUD (1): 30 SUD (2): 30 CG: 30	Alcohol (1), Heroin (2)	SUD (1): 32.8 ± 5.50 SUD (2): 29.1 ± 4.60 CG: 32.6 ± 4.90	SUD (1): 0/30 SUD (1): 0/30 CG: 0/30	mean LI for AS: right: 3.95, left: 2.80; Heroin right: 4.29, left: 2.22; CG right: 4.34, left: 2.08	Out-patient hospital treatment patients	Side-bias questionnaire with 5 items	↑ R bias heroin + CG; no preference in alcohol
	Weinland et al., 2019	SUD: 200 CG: 240	Alcohol	SUD: 48 CG: 48	SUD: 87/113 CG: 107/133	AS ♂: 10/7/85; AS ♀: 3/6/69; HC ♂: 8/11/114; HC ♀: 4/11 (A)/92	Semistructured interviews, ICD-10 + DSM-5 criteria met	Hole-in-the-card test	↔ eye preference
Dichotic listening	Drake et al., 1990	SUD: 25 CG: 25	Alcohol		SUD: 10/15 CG:10/15	All RH	Khavari Alcohol Test, met DSM-III-R criteria	Verbal + Musical task	Verbal: AS: ↑ R ear advantage; Musical: ↑ L ear advantage
	Ellis, 1990	SUD: 22 CG: 22	Alcohol	SUD: 46.1 ± 12.0 CG: 46.5 ± 13.0	SUD: 0/22 CG: 0/22	All RH	DIS, DSM-III criteria met	Verbal + Tone task	↔
	Hahn et al., 2010	SUD: 43 CG: 47	Nicotine		SUD: 21/22; CG: 27/20	All RH	FTND ≥1	consonant-vowel syllable task	All: ↑ R ear advantage; ↓ scores in ♂; ♂: ↓ LI
	Wasserman et al., 1999	87	multiple	9.0 ± 1.6	0/87	*Not specified*	DISC 2.3	consonant–vowel task	↓ R ear advantage predictive of substance use
Earedness	Mandal, 2000	SUD (1): 30 SUD (2): 30 CG: 30	Alcohol (1), Heroin (2)	SUD (1): 32.8 ± 5.50; SUD (2): 29.1 ± 4.60; CG: 32.6 ± 4.90	SUD (1): 0/30; SUD (1): 0/30; CG: 0/30	mean LI for AS: right: 3.95, left: 2.80; Heroin right: 4.29, left: 2.22; CG right: 4.34, left: 2.08	Out-patient hospital treatment patients	Side-bias questionnairewith 5 items	↑ R bias heroin + CG; no preference in alcohol
Handedness	Bakan, 1973	AS: 47	Alcohol	44	SUD: 0/47	7/5/35	Inpatients	handwriting selfreport	↑ LH (15 %) + ↑ NRH (25 %)
	Smith & Chyatte, 1983	AS: 64	Alcohol	19–53	SUD: 13/51	25/39	Former inpatients, actively involved in Alcoholics Anonymou	handwriting selfreport	↑ LH (39 %)
	London, 1986	SUD: 320	Alcohol (85 %)	*Not specified* (adults)	85/235	♂ 32/203 ♀ 10/75	Inpatients	EHI	♂: 14 % LH; ♀: 12 % LH
	McNamara et al., 1994	SUD: 43; At risk: 70 CG: 311	Alcohol	SUD: 41.3 CG: 19.0	SUD: 19/24; Risk: 31/39; CG: 253/58	♂ AS: 9/15 ♂ Risk 8/31 ♂ CG: 14/44 ♀ AS: 6/13 ♀ Risk: 4/27 ♀ CG: 39/214	Drinking inventory, recruited from Alcoholics Anonymous meeting	EHI	♂ ↑ LH (CG: 14.4 %, risk: 20.0 %, AS: 36.8 %) ♀ ↑ LH (CG: 13.7 %, risk: 12.9 %, AS: 31.5 %)
	Bíro & Novotny, 1992	SUD: 49	Alcohol	31.2–52.4	*Not specified*	*Not specified*	Inpatients	Finger tapping (speed)	30.6 % LH; 53.1 % RH; 16.3 % both hands
	Bíro, 1998	SUD: 25; CG: 25	Heroin	SUD: 17.4–21.3; CG: 17.8–18.2	SUD: 11/14 CG: 12/13	All RH according to self-report	Inpatients	Finger tapping (# taps) + Tracing task	↑ motor performance in RH
	Sperling, 2000; Sperling et al., 2010	SUD: 250; CG: 250	Alcohol	SUD: 43.8 ± 23.4; CG: age-matched	SUD: 125/125; CG: 125/125	AS: 63/187; CG: 29/221	Inpatients, DSM-III-R criteria met	Shimizu questionnaire, 13-items	♂: ↑ NRN (44 %); ♀: ↔
	Weinland et al., 2019	SUD: 200; CG: 240	Alcohol	SUD: 48; CG: 48	SUD: 87/113; CG: 107/133	AS ♂: 10/7/85; AS ♀: 3/6/69; CG ♂: 8/11/114; CG ♀: 4/11 (A)/92	Semistructured interviews, ICD-10 + DSM-5 criteria met	Shimizu questionnaire, 13-items	↔
	Mandal, 2000	SUD (1): 30; SUD (2): 30; CG: 30	Alcohol (1), Heroin (2)	SUD (1): 32.8 ± 5.50; SUD (2): 29.1 ± 4.60; CG: 32.6 ± 4.90	SUD (1): 0/30; SUD (1): 0/30; CG: 0/30	mean LI for AS: right: 3.95, left: 2.80; Heroin right: 4.29, left: 2.22; CG right: 4.34, left: 2.08	Out-patient hospital treatment patients	Side-bias questionnaire with 22 items, LI	↑ R bias heroin + CG; no preference in alcohol
Footedness	Mandal, 2000	SUD (1): 30; SUD (2): 30; CG: 30	Alcohol (1), Heroin (2)	SUD (1): 32.8 ± 5.50; SUD (2): 29.1 ± 4.60; CG: 32.6 ± 4.90	AS: 0/30; Heroin: 0/30; CG: 0/30	mean LI for AS: right: 3.95, left: 2.80; Heroin right: 4.29, left: 2.22; CG right: 4.34, left: 2.08	Out-patient hospital treatment patients	Side-bias questionnaire with 5 items	↑ R bias heroin + CG; no preference in alcohol

AS: Alcohol-addicted subjects.

CG: Control group.

DIS: Diagnostic Interview Schedule.

DISC-2.3: Diagnostic Interview Schedule for Children, Version 2.3.

FTND: Fagerström Test of Nicotine Dependence.

LH: Left-hander.

LI: Laterality index.

MA: Methamphetamine.

MH: Mixed-hander.

NRH: Non-right-handedness.

RH: Right-hander.

## Data Availability

No data was used for the research described in the article.

## Appendix A. Supplementary data

Supplementary data to this article can be found online at https://doi.org/10.1016/j.cpr.2025.102658.

## Abbreviations:

ACC: Anterior cingulate cortex
AD: Alcohol dependency
AIC: Anterior Insular Cortex
dlPFC: Dorsolateral Prefrontal Cortex
DTI: Diffusion tensor magnetic resonance imaging
EHI: Edinburgh Handedness Inventory
EEG: Electroencephalography
FA: Fractional anisotropy
fMRI: functional magnetic resonance imaging
FPN: Frontoparietal network
ICA: Independent component analysis
MRI: Magnetic resonance imaging
PFC: Prefrontal Cortex
SUD: Substance Use Disorder
vlPFC: Ventrolateral Prefrontal Cortex
vmPFC: Ventromedial Prefrontal Cortex
VBM: Voxel-based morphometry
